# Evaluation of the Implementation of Multiple Enhanced Recovery After Surgery Pathways Across a Provincial Health Care System in Alberta, Canada

**DOI:** 10.1001/jamanetworkopen.2021.19769

**Published:** 2021-08-06

**Authors:** Gregg Nelson, Xiaoming Wang, Alison Nelson, Peter Faris, Laura Lagendyk, Tracy Wasylak, Oliver F. Bathe, David Bigam, Erin Bruce, W. Donald Buie, Michael Chong, Adrian Fairey, M. Eric Hyndman, Anthony MacLean, Michael McCall, Sophia Pin, Haili Wang, Leah Gramlich

**Affiliations:** 1 Department of Oncology, University of Calgary, Calgary, Alberta, Canada; 2 Department of Obstetrics & Gynecology, University of Calgary, Calgary, Alberta, Canada; 3 Analytics, Data Integration, Measurement, and Reporting, Alberta Health Services, Calgary, Alberta, Canada; 4 Surgery Strategic Clinical Network, Alberta Health Services, Calgary, Alberta, Canada; 5 Alberta Health Services, Calgary, Alberta, Canada; 6 Strategic Clinical Networks, Alberta Health Services, Calgary, Alberta, Canada; 7 Department of Surgery, University of Calgary, Calgary, Alberta, Canada; 8 Department of Surgery, University of Alberta, Edmonton, Alberta, Canada; 9 Department of Anesthesiology, Perioperative and Pain Medicine, University of Calgary, Calgary, Alberta, Canada; 10 Department of Obstetrics & Gynecology, University of Alberta, Edmonton, Alberta, Canada; 11 Department of Medicine, University of Alberta, Edmonton, Alberta, Canada

## Abstract

**Importance:**

Engaging multidisciplinary care teams in surgical practice is important for the improvement of surgical outcomes.

**Objective:**

To evaluate the association of multiple Enhanced Recovery After Surgery (ERAS) pathways with ERAS guideline adherence and outcomes.

**Design, Setting, and Participants:**

This quality improvement study compared a pre-ERAS cohort (2013-2017) with a post-ERAS cohort (2014-2018). All patients were from Alberta Health Services in Alberta, Canada, and had available ERAS and up to 1-year postsurgery administrative data. Data collected included age, sex, body mass index, tobacco and alcohol use, diabetes, comorbidity index, and surgical characteristics. Data analysis was performed from May 7, 2020, to February 1, 2021.

**Interventions:**

Implementation of 5 ERAS pathways (colorectal, liver, pancreas, gynecologic oncology, and radical cystectomy) across 9 sites.

**Main Outcomes and Measures:**

Adherence to ERAS guidelines was measured by the percentage of patients whose care met the common ERAS pathway care element criteria. Surgical procedures were grouped by complexity; complications were classified by severity. Outcome measures for the pre-post–ERAS cohorts included length of stay (LOS), readmission, complications, and mortality.

**Results:**

A total of 7757 patients participated in the study, including 984 in the pre-ERAS cohort (median [interquartile range] age, 62 [53-71] years; 526 [53.5%] female) and 6773 in the post-ERAS cohort (median [interquartile range] age, 62 [53-71] years; 3470 [51.2%] male). In the total cohort, care-element adherence improved from 52% to 76% (*P* < .001), no significant differences were found in serious complications (from 6.2% to 4.9%; *P* = .08) or 30-day mortality (from 0.71% to 0.93%; *P* = .50), 1-year mortality decreased from 7.1% to 4.6% (*P* < .001), mean (SD) LOS decreased from 9.4 (7.0) to 7.8 (5.0) days (*P* < .001), and 30-day readmission rates were unchanged (from 13.4% to 11.7%; *P* = .12). After adjustment for patient characteristics, the LOS mean difference decreased 0.71 days (95% CI, −1.13 to −0.29 days; *P* < .001), with no significant differences in adjusted 30-day readmission (−3.5%; 95% CI, −22.7% to 20.4%; *P* = .75), serious complications (1.3%; 95% CI, −26.2% to 39.0%; *P* = .94), or mortality (30-day mortality: 42% [95% CI, −35.4% to 212.3%]; *P* = .38; 1-year mortality: 8% [95% CI, −20.5% to 46.8%]; *P* = .62). The adjusted 1-year readmission rate was −15.6% (95% CI, −27.7% to −1.5%; *P* = .03) in favor of ERAS, and readmission LOS was shorter by 1.7 days (95% CI, −3.3 to −0.1 days; *P* = .04).

**Conclusions and Relevance:**

The results of this quality improvement study suggest that implementation of ERAS across multiple pathways may improve health care practitioner adherence to ERAS guidelines, LOS, and readmission rates at a system level.

## Key Points

**Question:**

Does the implementation of multiple Enhanced Recovery After Surgery (ERAS) pathways across 1 health care system affect ERAS guideline adherence, length of stay, complications, readmissions, and mortality?

**Findings:**

In this quality improvement study, comparison of pre-post–ERAS cohorts found that pathway implementation was associated with increased guideline adherence, from 52% to 76%; decreased unadjusted length of stay, from a mean of 9.4 to 7.8 days; and decreased adjusted length of stay by 0.71 days, with no differences in serious complications or 30-day mortality.

**Meaning:**

These results suggest that implementation of multiple ERAS pathways could provide system-level improvement in ERAS guidelines adherence and length of stay.

## Teaser

This quality improvement study evaluates the implementation of Enhanced Recovery After Surgery pathways to examine the association with guideline adherence, length of stay, complications, readmissions, and mortality.

## Introduction

Enhanced Recovery After Surgery (ERAS) is a global surgical quality improvement program that involves evidence-based guidelines and implementation strategies designed to improve patient outcomes by engaging multidisciplinary care teams in surgical practice change.^1^ An increasing body of evidence has demonstrated the effectiveness of systemwide implementation of ERAS in improving patient outcomes while achieving cost savings in colorectal surgery^2,3,4,5^ and other disciplines.^6^ Benefits include decreased hospital length of stay (LOS) and decreased complications, with no significant increase in readmission or mortality rates.^1^ Economic evaluation of ERAS has demonstrated its cost-effectiveness and supported expansion across surgical disciplines with return on investment ratios as high as 7.3.^7^

On the basis of this evidence, Alberta Health Services (AHS) expanded the scope of its ERAS program to multiple surgical pathways and hospital sites with continued engagement of leadership and health care practitioners.^8^ Alberta Health Services is a publicly funded provincial health care system where approximately 288 000 operations are performed annually across 55 hospital sites.^9^ Since the launch of ERAS for colorectal surgery in AHS in 2013,^2^ ERAS practices have expanded across surgical disciplines and hospital sites throughout the province. The aim of this quality improvement study was to evaluate the association of implementing multiple ERAS pathways across a health care system with ERAS guideline adherence and outcomes (LOS, complications, readmissions, and mortality).

## Methods

Ethics approval for this study was obtained from the Conjoint Health Research Ethics Board of the University of Calgary, Alberta, Canada. Informed consent was not required for this quality improvement study because the research involved no more than minimal risk to the study participants, the waiver or alteration would not adversely affect the rights and welfare of the participants, and the research could not practicably (feasibly) be performed without the waiver. All data were deidentified. The study followed the Standards for Quality Improvement Reporting Excellence (SQUIRE) reporting guideline.^10^

### ERAS Implementation in Alberta

ERAS implementation in Alberta began with the colorectal surgery pathway at 6 sites from January 1, 2013, to December 31, 2015, to address variations in surgical outcomes, cost, and capacity pressures. Funding for ERAS was provided by AHS’ Strategic Clinical Networks^11^ to support implementation and resource development. On the basis of demonstrated improvements at the initial sites^2,3^ and identified barriers and facilitators to implementation specific to the Alberta context,^12^ the ERAS approach was redesigned. From January 1, 2016, to December 31, 2018, additional AHS funding was provided to implement ERAS guidelines across multiple surgical pathways and sites to improve adherence with ERAS guidelines and clinical and system outcomes. With the use of a structured approach, flexible implementation steps were developed to guide local site teams from preimplementation to sustainable ERAS practices during an 18-month period. Implementation activities focused on clinical and operational leadership, clinical best practices alignment, and development of robust intersite relationships. Expansion of ERAS to a new site or pathway was driven by surgery, anesthesia, and operational leadership readiness. This study reports on the quality improvement results of ERAS pathway implementation in 5 areas of surgery (colorectal, liver, pancreas, gynecologic oncology, and radical cystectomy) across 9 sites (Figure).

**Figure.**
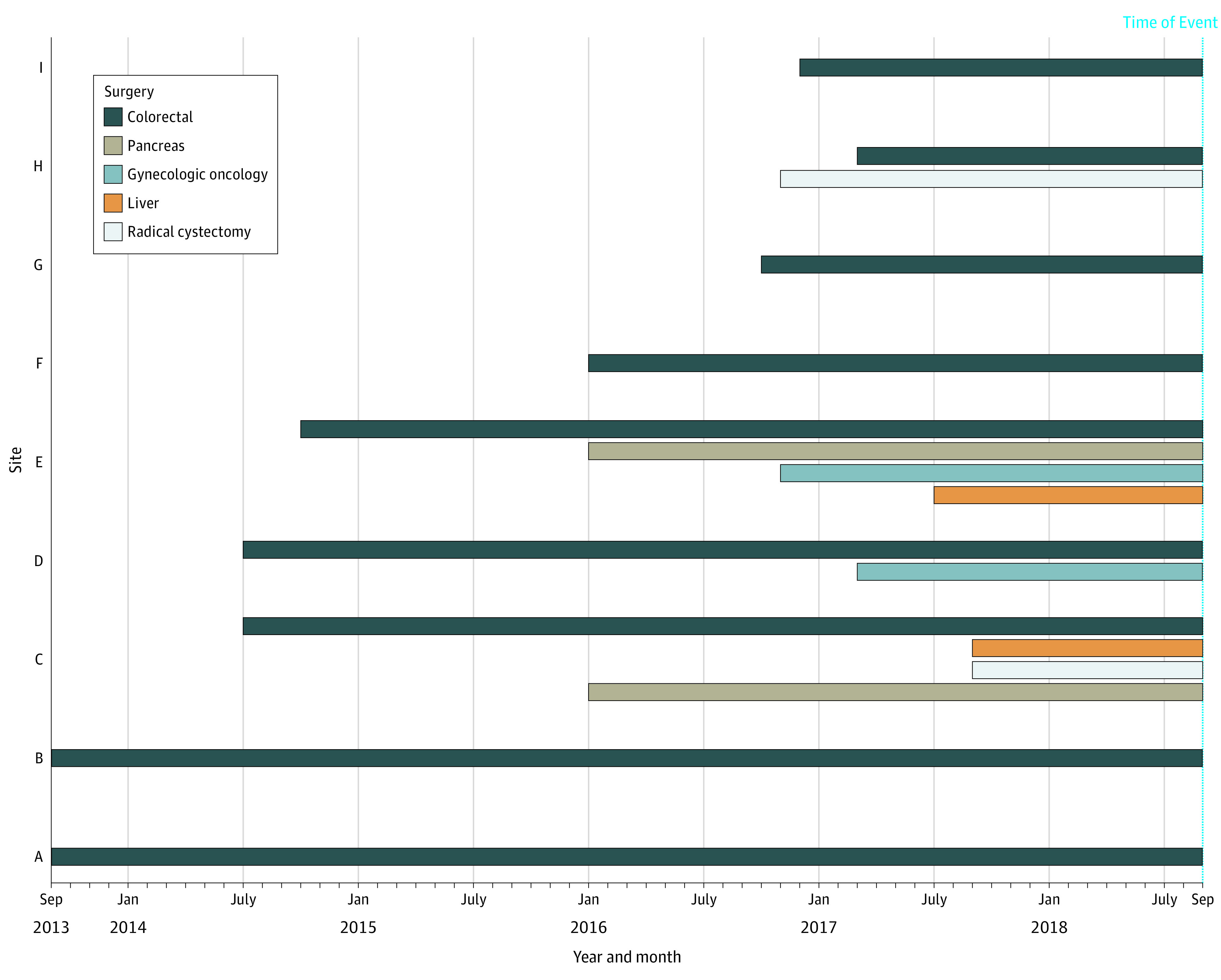
Staged Enhanced Recovery After Surgery Pathway Implementation Timelines and Sites per Pathway

### Outcome Measures

#### ERAS Guideline Elements and Adherence Measures

ERAS guidelines and care elements vary by phase of care and surgery pathway.^13,14,15,16,17^ For a description of the specific care elements included in each ERAS pathway in this study, see the AHS website,^18^ including the ERAS Alberta Care Standards for each of the 5 pathways, which are available in eAppendix 1 in the Supplement.

The defined ERAS care elements were extracted from clinical records after surgery and after patient discharge and entered into the ERAS Interactive Audit System (EIAS; Encare AB) following AHS ERAS detailed data collection definitions. A set of ERAS care elements common across all pathways in this study was identified and grouped by phase of care (ie, preadmission, preoperative, intraoperative, and postoperative). The specific elements included within each phase of care are provided in eAppendix 2 in the Supplement.

Adherence in this study was measured by the percentage of eligible patients who met the criteria for a given care element and by the total percentage of eligible patients who met the criteria within a phase of care. Total adherence for multiple ERAS pathways is a summary of adherence for the common ERAS care elements across pathways for the total cohort. Total adherence within a pathway represents adherence for all care elements captured for patients within that pathway (ie, the common ERAS care elements plus any additional elements for that pathway).

#### Patient Data, Cohorts, and Outcome Measures

In this pre-post comparison, patient baseline data were all collected retrospectively by medical record review and entered into the EIAS, aiming for at least 50 consecutive patients at each site who met the inclusion criteria for the ERAS pathway during a 3- to 6-month period, depending on site volumes.^19^ These patients formed the pre-ERAS cohort with standardized AHS ERAS data collection to help inform the priority activities for site and pathway implementation. Surgical procedures were grouped by complexity: low (surgically less complex) or medium to high (surgically more complex)^2^ (eAppendix 3 in the Supplement). Complications were classified according to Dindo et al,^20^ with serious complications defined as grade IIIA or higher (on a scale of I to V [I, II, IIIA, IIIB, IVA, IVB, V], with V indicating greatest severity). Administrative data after discharge were extracted from the Discharge Abstract Database. Outcome measures for the pre- and post-ERAS cohorts gathered from the Discharge Abstract Database included the following: LOS (number of days between primary operation and discharge), readmission (patients readmitted to an Alberta acute care facility within 30 days and 1 year of discharge), and mortality (30-day and 1-year all-cause mortality). The post-ERAS cohort included all ERAS patients with EIAS data and Discharge Abstract Database data collected for the study period of September 1, 2013, to September 31, 2018.

### Statistical Analysis

Continuous variables were summarized using mean (SD) and median (interquartile range [IQR]). For categorical variables, number (percentage) was provided. Nonparametric Wilcoxon tests and χ^2^ tests were used to compare the difference between pre- and post-ERAS characteristics for continuous and categorical variables, respectively. Data analysis was performed from May 7, 2020, to February 1, 2021.

The LOS outcome measures were analyzed using negative binomial regression.^21^ The coefficient for the exposure variable was expressed as the percentage change in LOS of patients in the postimplementation period compared with those in the preimplementation period. The binary outcome variables of readmission within 30 days after discharge and serious complications were analyzed using logistic regression. Marginal mean differences in LOS were calculated from the negative binomial regression models using the margins procedure in R, version 0.3.25 (R Foundation for Statistical Computing).^22^

Multivariate models were adjusted for potential confounding factors, including age, sex, body mass index (BMI; calculated as weight in kilograms divided by height in meters squared), procedure group, surgical complexity, tobacco and alcohol use (yes/no variables), Charlson Comorbidity Index (CCI),^23^ American Society of Anesthesiologists (ASA) class, diabetes, final diagnosis (benign or malignant), and implementation site. Stepwise variable selection was used for creating parsimonious models. Variables were included in the model if the probability value for their association was a 2-sided *P* < .05.

## Results

### Patient Characteristics

A total of 7757 patients participated in the study, including 984 in the pre-ERAS cohort (median [interquartile range] age, 62 [53-71] years; 526 [53.5%] female) and 6773 in the post-ERAS cohort (median [interquartile range] age, 62 [53-71] years; 3470 [51.2%] male). Summary demographic characteristics are given in Table 1. Within the total cohort, the distribution of patients in the pre-ERAS vs post-ERAS cohorts was not significantly different according to age (≤50 years of age: 195 [19.8%] vs 1403 [20.7%]; 51-64 years of age: 364 [37.0%] vs 2510 [37.1%]; 65-75 years of age: 272 [27.6%] vs 1957 [28.9%]; >75 years of age: 153 [15.6%] vs 903 [13.3%]; *P* = .27), BMI (BMI <25: 334 [33.9%] vs 2295 [33.9%]; BMI 25-29.9: 346 [35.2%] vs 2234 [33.0%]; BMI ≥30: 304 [30.9%] vs 2244 [33.1%]; *P* = .28), tobacco use (182 [18.5%] vs 1373 [20.3%]; *P* = .19), and diabetes (61 [6.2%] vs 337 [5.0%]; *P* = .10). Significant differences were found in distributions of patients in the pre-ERAS vs post-ERAS cohorts in the total cohort for sex (female: 526 [53.5%] vs 3303 [48.8%]; *P* = .006), alcohol use (475 [48.3%] vs 3743 [55.3%]; *P* < .001), CCI (CCI 0: 80 [8.1%] vs 885 [13.1%]; CCI 1: 300 [30.5%] vs 2508 [37%]; CCI ≥2: 604 [61.4%] vs 3380 [49.9%]; *P* < .001), surgical characteristics (ASA class 1-2: 605 [63.2%] vs 4612 [69.4%]; ASA class 3-5: 352 [36.8%] vs 2037 [30.6%]; *P* < .001; low complexity: 372 [37.8%] vs 3619 [53.4%]; high complexity: 612 [62.2%] vs 3154 [46.6%]; *P* < .001; and final diagnosis of benign: 715 [72.7%] vs 6118 [90.3%]; final diagnosis of malignant: 269 [27.3%] vs 655 [9.7%]; *P* < .001), and site of implementation (Chinook Regional Hospital: 50 [26.9%] vs 136 [73.1%]; Foothills Medical Centre: 285 [17.6%] vs 1334 [82.4%]; Grey Nuns Community Hospital: 49 [5.7%] vs 809 [94.3%]; Misericordia Community Hospital: 44 [7.7%] vs 525 [92.3%]; Peter Lougheed Centre: 66 [4.1%] vs 1550 [95.9%]; Royal Alexandra Hospital: 63 [7.0%] vs 832 [93.0%]; Red Deer Regional: 50 [19.1%] vs 212 [80.9%]; Rockyview General Hospital: 99 [36.1%] vs 175 [63.9%]; University of Alberta Hospital: 278 [18.8%] vs 1200 [81.2%]; *P* < .001). Significant differences in distribution of some patient characteristics were found within specific surgical pathways for the pre-ERAS vs post-ERAS cohorts (colorectal pathway: ≤50 years of age: 88 [18.5%] vs 1145 [20.3%]; 51-64 years of age: 188 [39.4%] vs 2061 [36.5%]; 65-75 years of age: 119 [25.0%] vs 1651 [29.3%]; >75 years of age: 82 [17.2%] vs 786 [13.9%]; *P* = .049; final diagnosis of benign: 456 [95.6%] vs 5500 [97.5%]; final diagnosis of malignant: 21 [4.4%] vs 143 [2.5%]; *P* = .02; liver pathway: ASA class 1-2: 55 [56.1%] vs 92 [70.2%]; ASA class 3-5: 43 [43.9%] vs 39 [29.8%]; *P* = .03; pancreas pathway: BMI <25: 58 [37.4%] vs 105 [36.7%]; BMI 25-29.9: 68 [43.9%] vs 98 [34.3%]; BMI ≥30: 29 [18.7%] vs 83 [29.0%]; *P* = .04; alcohol use: 52 [33.6%] vs 62 [21.7%]; *P* = .007; low procedure complexity: 37 [23.9%] vs 101 [35.3%]; high procedure complexity: 118 [76.1%] vs 185 [64.7%]; *P* = .01; gynecologic oncology pathway: alcohol use: 74 [48.7%] vs 400 [67.7%]; *P* < .001; CCI 0: 0 vs 2 [0.3%]; CCI 1: 53 [34.9%] vs 293 [49.6%]; CCI ≥2: 99 [65.1%] vs 296 [50.1%]; *P* = .004; low procedure complexity: 76 [50.0%] vs 460 [77.8%]; high procedure complexity: 76 [50.0%] vs 131 [22.2%]; *P* < .001; and final diagnosis of benign: 43 [28.3%] vs 271 [45.9%]; final diagnosis of malignant: 109 [71.7%] vs 320 [54.2%]; *P* < .001).

**Table 1.** Patient Characteristics in the Pre- and Post-ERAS Cohorts for Multiple ERAS Pathways and Specific Pathways

Measure	No. (%) of patients																	
	Multiple ERAS pathways			Colorectal			Liver			Pancreas			Gynecologic oncology			Radical cystectomy		
	Pre (n = 984)	Post (n = 6773)	*P* value	Pre (n = 477)	Post (n = 5643)	*P* value	Pre (n = 99)	Post (n = 131)	*P* value	Pre (n = 155)	Post (n = 286)	*P* value	Pre (n = 152)	Post (n = 591)	*P* value	Pre (n = 101)	Post (n = 122)	*P* value
Age category, y																		
≤50	195 (19.8)	1403 (20.7)	.27	88 (18.5)	1145 (20.3)	.049	37 (37.4)	37 (28.2)	.52	30 (19.4)	53 (18.5)	.11	35 (23.0)	165 (27.9)	.39	5 (5.0)	3 (2.5)	.74
51-64	364 (37.0)	2510 (37.1)		188 (39.4)	2061 (36.5)		32 (32.3)	46 (35.1)		49 (31.6)	103 (36.0)		65 (42.8)	262 (44.3)		30 (29.7)	38 (31.2)	
65-75	272 (27.6)	1957 (28.9)		119 (25.0)	1651 (29.3)		23 (23.2)	36 (27.5)		50 (32.3)	104 (36.4)		35 (23.0)	114 (19.3)		45 (44.6)	52 (42.6)	
>75	153 (15.6)	903 (13.3)		82 (17.2)	786 (13.9)		7 (7.1)	12 (9.2)		26 (16.8)	26 (9.1)		17 (11.2)	50 (8.5)		21 (20.8)	29 (23.8)	
Sex																		
Male	458 (46.5)	3470 (51.2)	.006	257 (53.9)	3163 (56.1)	.36	43 (43.4)	69 (52.7)	.17	76 (49.0)	144 (50.4)	.79	0	0	NA	82 (81.2)	94 (77.1)	.45
Female	526 (53.5)	3303 (48.8)		220 (46.1)	2480 (44.0)		56 (56.6)	62 (47.3)		79 (51.0)	142 (49.7)		152 (100)	591 (100)		19 (18.8)	28 (23)	
BMI category																		
<25	334 (33.9)	2295 (33.9)	.28	161 (33.8)	1939 (34.4)	.53	31 (31.3)	50 (38.2)	.11	58 (37.4)	105 (36.7)	.04	50 (32.9)	159 (26.9)	.08	34 (33.7)	42 (34.4)	.80
25-29.9	346 (35.2)	2234 (33.0)		152 (31.9)	1900 (33.7)		41 (41.4)	37 (28.2)		68 (43.9)	98 (34.3)		47 (30.9)	158 (26.7)		38 (37.6)	41 (33.6)	
≥30	304 (30.9)	2244 (33.1)		164 (34.4)	1804 (32.0)		27 (27.3)	44 (33.6)		29 (18.7)	83 (29.0)		55 (36.2)	274 (46.4)		29 (28.7)	39 (32.0)	
Tobacco use	182 (18.5)	1373 (20.3)	.19	85 (17.8)	1157 (20.5)	.16	25 (25.3)	25 (19.1)	.26	27 (17.4)	51 (17.8)	.91	19 (12.5)	102 (17.3)	.16	26 (25.7)	38 (31.2)	.37
Alcohol use	475 (48.3)	3743 (55.3)	<.001	279 (58.5)	3190 (56.5)	.40	35 (35.4)	56 (42.8)	.26	52 (33.6)	62 (21.7)	.007	74 (48.7)	400 (67.7)	<.001	35 (34.7)	35 (28.7)	.34
Charlson Comorbidity Index																		
0	80 (8.1)	885 (13.1)	<.001	78 (16.4)	875 (15.5)	.497	1 (1.0)	5 (3.8)	.07	1 (0.7)	3 (1.1)	.89	0	2 (0.3)	.004	0	0	.16
1	300 (30.5)	2508 (37.0)		163 (34.2)	2081 (36.9)		10 (10.1)	5 (3.8)		51 (32.9)	91 (31.8)		53 (34.9)	293 (49.6)		23 (22.8)	38 (31.2)	
≥2	604 (61.4)	3380 (49.9)		236 (49.5)	2687 (47.6)		88 (88.9)	121 (92.4)		103 (66.5)	192 (67.1)		99 (65.1)	296 (50.1)		78 (77.2)	84 (68.9)	
ASA class																		
1-2	605 (63.2)	4612 (69.4)	<.001	319 (70.3)	3864 (69.9)	.87	55 (56.1)	92 (70.2)	.03	83 (54.3)	159 (56.6)	.64	103 (67.8)	436 (74.3)	.11	45 (45.0)	61 (50.0)	.46
3-5	352 (36.8)	2037 (30.6)		135 (29.7)	1664 (30.1)		43 (43.9)	39 (29.8)		70 (45.8)	122 (43.4)		49 (32.2)	151 (25.7)		55 (55.0)	61 (50.0)	
Procedure complexity score class																		
Low	372 (37.8)	3619 (53.4)	<.001	248 (52.0)	3049 (54.0)	.39	11 (11.1)	9 (6.9)	.26	37 (23.9)	101 (35.3)	.01	76 (50.0)	460 (77.8)	<.001	0	0	NA
High	612 (62.2)	3154 (46.6)		229 (48.0)	2594 (46.0)		88 (88.9)	122 (93.1)		118 (76.1)	185 (64.7)		76 (50.0)	131 (22.2)		101 (100)	122 (100)	
Final diagnosis																		
Benign	715 (72.7)	6118 (90.3)	<.001	456 (95.6)	5500 (97.5)	.02	73 (73.7)	95 (72.5)	.84	138 (89)	240 (83.9)	.14	43 (28.3)	271 (45.9)	<.001	5 (5.0)	12 (9.8)	.17
Malignant	269 (27.3)	655 (9.7)		21 (4.4)	143 (2.5)		26 (26.3)	36 (27.5)		17 (11)	46 (16.1)		109 (71.7)	320 (54.2)		96 (95.1)	110 (90.2)	
Diabetes	61 (6.2)	337 (5.0)	.10	29 (6.1)	284 (5.0)	.32	5 (5.1)	4 (3.1)	.44	13 (8.4)	15 (5.2)	.20	6 (4.0)	18 (3.1)	.57	8 (7.9)	16 (13.1)	.21

Abbreviations: ASA, American Society of Anesthesiologists; BMI, body mass index (calculated as weight in kilograms divided by height in meters squared); ERAS, Enhanced Recovery After Surgery; NA, not applicable.

### Adherence Measures

Total adherence (all pathways and all phases, N = 7757) improved from 52% in the pre-ERAS cohort to 76% in the post-ERAS cohort (*P* < .001) (Table 2). The greatest gains in adherence were found in the preadmission phase (13% in the pre-ERAS cohort to 63% in the post-ERAS cohort) and postoperative phase of ERAS (33% in the pre-ERAS cohort to 57% in the post-ERAS cohort) with moderate gains in preoperative phase adherence (69% in the pre-ERAS cohort to 83% in the post-ERAS cohort) (*P* < .001).

**Table 2.** ERAS Care Adherence in the Pre- and Post-ERAS Cohorts for Multiple ERAS Pathways and Specific Pathways

Measure	Adherence, %																	
	Multiple ERAS pathways			Colorectal			Liver			Pancreas			Gynecologic oncology			Radical cystectomy		
	Pre (n = 984)	Post (n = 6773)	*P* value	Pre (n = 477)	Post (n = 5643)	*P* value	Pre (n = 99)	Post (n = 131)	*P* value	Pre (n = 155)	Post (n = 286)	*P* value	Pre (n = 152)	Post (n = 591)	*P* value	Pre (n = 101)	Post (n = 122)	*P* value
Total	52.12	75.81	<.001	41.78	67.48	<.001	57.88	65.78	<.001	44.43	66.02	<.001	53.37	75.25	<.001	38.48	61.64	<.001
Preadmission	12.62	63.41	<.001	6.51	62.61	<.001	48.75	67.31	<.001	18.04	69.44	<.001	16.67	67.48	<.001	3.44	63.29	<.001
Preoperative	69.00	83.33	<.001	62.60	82.26	<.001	78.53	84.11	<.001	67.78	86.26	<.001	78.66	89.05	<.001	68.76	86.97	<.001
Intraoperative	89.79	95.53	<.001	69.17	77.16	<.001	63.85	64.43	<.001	65.04	73.97	<.001	76.98	79.53	<.001	57.67	56.88	<.001
Postoperative	32.92	57.29	<.001	23.37	52.05	<.001	31.29	47.31	<.001	21.60	41.24	<.001	29.67	62.17	<.001	14.69	41.26	<.001

Abbreviation: ERAS, Enhanced Recovery After Surgery.

Improvement in ERAS adherence was exhibited in all surgical pathways when measured as total adherence within the pathway and at all phases of care. The greatest changes in total adherence within a surgical pathway were found in the colorectal pathway (42% in the pre-ERAS cohort to 67% in the post-ERAS cohort; *P* < .001). Increases in total adherence were comparable for the pathways for radical cystectomy (38% in the pre-ERAS cohort to 62% in the post-ERAS cohort; *P* < .001), gynecologic oncology (53% in the pre-ERAS cohort to 75% in the post-ERAS cohort; *P* < .001), and pancreas surgery (44% in the pre-ERAS cohort to 66% in the post-ERAS cohort; *P* < .001). The liver surgery pathway had the lowest improvement in total adherence, from 58% in the pre-ERAS cohort to 66% in the post-ERAS cohort (*P* < .001).

### LOS, Readmission, and Serious Complications Rates

For the total pre-post cohort, the unadjusted LOS decreased from a mean (SD) of 9.4 (10.3) days (median, 7 days; IQR, 5-11 days) in the pre-ERAS cohort to a mean (SD) of 7.8 (10.3) days (median, 5 days; IQR, 4-8 days) in the post-ERAS cohort (*P* < .001) (Table 3). No statistically significant differences were found for serious complications (from 6.2% to 4.9%; *P* = .08) or 30-day mortality (from 0.71% to 0.93%; *P* = .50); however, 1-year mortality decreased from 7.1% in the pre-ERAS cohort to 4.6% in the post-ERAS cohort (*P* < .001). Although 30-day readmission rates were unchanged, in those patients who were readmitted, the LOS was shorter in the post-ERAS cohort (mean [SD], 7.6 [13.2] days) compared with the pre-ERAS cohort (mean [SD], 9.8 [11.5] days; *P* = .007).

**Table 3.** Patient Length of Stay, Readmission, and Serious Complication Rates for the Pre- and Post-ERAS Cohorts for Multiple ERAS Pathways and Specific Pathways

Measure	Multiple ERAS pathways			Colorectal			Liver			Pancreas			Gynecologic oncology			Radical cystectomy		
	Pre (n = 984)	Post (n = 6773)	*P* value	Pre (n = 477)	Post (n = 5643)	*P* value	Pre (n = 99)	Post (n = 131)	*P* value	Pre (n = 155)	Post (n = 286)	*P* value	Pre (n = 152)	Post (n = 591)	*P* value	Pre (n = 101)	Post (n = 122)	*P* value
Length of stay acute, d																		
Mean (SD)	9.41 (10.31)	7.76 (10.27)	<.001	8.59 (8.83)	7.73 (10.24)	<.001	8.69 (10.17)	7.24 (5.01)	.61	13.47 (15.08)	14.26 (14.70)	.74	5.74 (6.75)	3.85 (4.88)	<.001	13.26 (9.47)	13.16 (12.19)	.03
Median (IQR)	7 (5-11)	5 (4-8)		6 (4-10)	5 (4-8)		6 (5-9)	6 (5-8)		10 (7-14)	10 (7-16)		4 (2-6)	3 (2-4)		12 (8-15)	9 (7-14)	
30-d Readmission rate, %	13.41	11.71	.12	12.37	11.64	.64	7.07	9.92	.45	23.23	20.28	.47	6.58	5.92	.76	19.80	24.59	.39
Serious complications rate, %	6.20	4.90	.08	4.19	4.80	.55	11.11	3.82	.03	11.61	11.54	.98	3.29	1.86	.28	6.93	9.84	.44
30-d Mortality, %	0.71	0.93	.498	0.42	0.92	.26	0	0.76	.38	1.29	1.05	.82	0	0.68	.31	2.97	2.46	.81
1-y Mortality, %	7.11	4.55	<.001	3.56	3.86	.74	5.05	2.29	.26	13.55	15.38	.60	6.58	3.89	.15	16.83	16.39	.93

Abbreviations: ERAS, Enhanced Recovery After Surgery; IQR, interquartile range.

Within the specific surgical pathways, unadjusted LOS reduction was observed for the colorectal (mean [SD], 8.6 [8.8] days; median [IQR], 6 [4-10] days for the pre-ERAS cohort vs 7.7 [10.2] days; median [IQR], 5 [4-8] days for the post-ERAS cohort; *P* < .001), gynecologic oncology (mean [SD], 5.7 [6.8] days; median [IQR], 4 [2-6] days in the pre-ERAS cohort vs 3.9 [4.9] days; median [IQR], 3 [2-4] days in the post-ERAS cohort; *P* < .001), and radical cystectomy (mean [SD], 13.3 [9.5] days; median [IQR], 12 [8-15] days in the pre-ERAS cohort vs 13.2 [12.2] days; median [IQR], 9 [7-14] days in the post-ERAS cohort; *P* = .03) cohorts. Serious complications were unchanged except in the liver cohort in which a reduction was observed (11.1% in the pre-ERAS cohort vs 3.8% in the post-ERAS cohort; *P* = .03). Although 30-day readmission rates did not significantly change (13.4% to 11.7%; *P* = .12), in patients undergoing colorectal surgery who were readmitted, the LOS was shorter in the post-ERAS cohort (mean [SD], 7.8 [14.3] days) compared with the pre-ERAS cohort (mean [SD], 10.3 [10.7] days; *P* = .01).

For the total pre-post cohort, the adjusted mean (SD) difference for LOS was −0.71 (0.22) days (*P* < .001) (ie, ERAS was associated with a 0.71-day decrease) (Table 4). Variables selected for the regression modeling are listed in the eTable in the Supplement). No differences were found for 30-day readmission (odds ratio [OR], −3.5; 95% CI, −22.7 to 20.4; *P* = .75), serious complications (OR, 1.3; 95% CI, −26.2 to 39.0; *P* = .94), or mortality after adjustment (30-day mortality: OR, 42.0; 95% CI, −35.4 to 212.3; *P* = .38; 1-year mortality: OR, 8.0; 95% CI, −20.5 to 46.8; *P* = .62). The relative change in 1-year readmission rate, however, for the total cohort was −15.6% in favor of ERAS (*P* = .03), and in those patients who required readmission, the ERAS cohort was admitted for 1.7 fewer days (*P* = .04).

**Table 4.** Changes in Patient Length of Stay, Readmission, and Serious Complication Rates, Adjusted for Significant Patient Characteristics for Multiple ERAS Pathways and Specific Pathways

Measure	Multiple ERAS pathways		Colorectal		Liver		Pancreas		Gynecologic oncology		Radical cystectomy	
	Rate (relative change in rate), % (n = 984 pre and 6773 post)	*P* value	Rate (relative change in rate), % (n = 477 pre and 564 post)	*P* value	Rate (relative change in rate), % (n = 99 pre and 131 post)	*P* value	Rate (relative change in rate), % (n = 155 pre and 286 post)	*P* value	Rate (relative change in rate), % (n = 152 pre and 591 post)	*P* value	Rate (relative change in rate), % (n = 101 pre and 122 post)	*P* value
Length of stay, acute adjusted mean difference, d	−0.71 (−1.13 to −0.29)	<.001	−1.05 (−1.62 to −0.47)	<.001	−1.25 (−2.49 to 0.00)	.05	2.75 (0.98 to 4.52)	.002	−0.59 (−1.15 to −0.03)	.04	−1.32 (−3.27 to 0.62)	.18
30-d Readmission, adjusted	−3.5 (−22.7 to 20.4)	.75	−3.3 (−28.4 to 30.6)	.83	31.1 (−50.4 to 246.3)	.58	−16.7 (−48.6 to 35.0)	.46	−18.1 (−60.7 to 70.5)	.59	32.9 (−30.6 to 154.8)	.39
1-y Readmission, adjusted	−15.6 (−27.7 to −1.5)	.03	−20.0 (−34.7 to −1.9)	.03	−34.4 (−63.5 to 17.7)	.16	−13.7 (−41.9 to 28.2)	.47	−2.6 (−37.5 to 51.7)	.91	6.9 (−37.5 to 82.8)	.81
Serious complication, adjusted	1.3 (−26.2 to 39.0)	.94	10.5 (−31.0 to 77.0)	.68	−72.9 (−91.3 to −15.3)	.02	70.4 (−15.1 to 242.1)	.13	−43.9 (−80.8 to 64.1)	.29	61.9 (−39.5 to 333.4)	.34
30-d Mortality	42.0 (−35.4 to 212.3)	.38	124.6 (−45.7 to 828.7)	.26			−18.5 (−86.5 to 393.0)	.82			−18.5 (−83.9 to 313.0)	.80
1-y Mortality	8.0 (−20.5 to 46.8)	.62	9.2 (−34.4 to 82.1)	.73	−56.4 (−89.8 to 87.0)	.26	35.9 (−23.8 to 142.4)	.30	−10.3 (−60.9 to 105.9)	.70	−0.7 (−52.5 to 107.6)	.99

Abbreviation: ERAS, Enhanced Recovery After Surgery.

Within the specific surgical pathways, adjusted LOS reduction was observed in the colorectal (−1.1 days; 95% CI, −1.62 to −0.47; *P* < .001) and gynecologic oncology (overall: −0.6 days [95% CI, −1.2 to −0.03 days]; high complexity: −1.5 days [95% CI, −3.0 to −0.1]; *P* = .04) cohorts. In the pancreas cohort, ERAS was associated with a prolongation of LOS of 2.8 days (95% CI, 1.0-4.5 days; *P* = .002). In the patients undergoing colorectal surgery and radical cystectomy who required readmission, the ERAS cohorts were admitted for 2.9 (95% CI, 0.1-5.6) fewer days in the colorectal surgery group and 3.4 (95% CI, 0.1-6.6) fewer days in the radical cystectomy group (*P* = .04). The only change in complications that was observed was in the liver cohort, where ERAS was associated with a 73% (95% CI, 15.3%-91.3%; *P* = .02) reduction in serious complications.

## Discussion

This study, to our knowledge the largest ERAS quality improvement study of its kind to date, found statistically significant improvements at the system level in ERAS adherence, LOS, and readmissions. Of importance, these changes appear to not have come at the expense of increased complications. Expansion of ERAS across multiple pathways and sites throughout AHS provided an opportunity to assess changes associated with systemwide implementation of ERAS. Although evidence supports ERAS practice changes^1^ and previous research has demonstrated the benefits of systemwide expansion of ERAS within a single surgical area,^2,5,6^ none to our knowledge have reported the impact of systemwide expansion of ERAS across multiple sites and disciplines.

Pickens et al^24^ examined the results of multidisciplinary audit of ERAS programs at a single institution. They studied clinical outcome improvements in aggregate and within specific surgical pathways (liver, pancreas, colorectal, and urology). A significant reduction in unadjusted LOS (1.5 days) associated with improved ERAS adherence was reported, together with improved 30-day survival across all specialties. In the present study, an aggregate reduction in LOS of 1.6 days in favor of ERAS and decreased 1-year mortality was found. After adjustment for confounders, however, the LOS reduction was 0.7 days, and the association with mortality was no longer significant. An important finding in this study was the association of ERAS with aggregate readmissions (15% decreased 1-year readmission rate and readmission LOS that was approximately 2 days shorter for ERAS patients). This finding is consistent with a previous meta-analysis^25^ that found a 20% reduction in readmission in favor of ERAS.

From a quality improvement perspective, the pathway-specific findings are worthy of discussion because this provides insight into where health care systems need to focus to gain further benefits from ERAS implementation. In 2016, an audit was performed of the ERAS colorectal program in 1333 patients across 6 sites in Alberta, which found a median 1.5-day reduction in LOS.^2^ Since then, the number of sites has been expanded to 9, and the ERAS colorectal cohort is now composed of 5643 patients. ERAS adherence has remained relatively stable (67%); however, this study found a 1-day reduction in LOS with a median LOS of 5 days, which is compatible with other large cohort ERAS studies.^26^ Although this finding could be considered acceptable sustainment given that clinical outcome improvements may diminish as health systems scale up interventions, the aim should be higher. It is established that there is an inverse dose-response association between ERAS adherence and clinical outcome improvements^27^ including LOS,^26^ a finding first confirmed in colorectal surgery. The ERAS Compliance Group found that increasing ERAS colorectal protocol adherence was correlated with shorter LOS, with the greatest improvements seen with adherence greater than 90%.^26^ Furthermore, Gustafsson et al^27^ reported that adverse postoperative outcomes were significantly reduced with increasing ERAS adherence starting at a threshold of 70%. These studies highlight that continued audit and feedback may be important in improving ERAS adherence. On the basis of the literature, this should translate to further improvements in clinical outcomes, particularly within our system, given that overall ERAS colorectal adherence has never exceeded 70%. In the early years of AHS ERAS colorectal implementation, multidisciplinary ERAS teams would audit perioperative practice biweekly by site, using the EIAS to examine ERAS adherence and outcomes^2^ and devise PDSA (plan-do-study-act) cycles targeted to areas of low adherence.^1^ These efforts resulted in an increase in adherence of 20% with commensurate improvement in clinical outcomes. In recent years, however, efforts have focused on the spread and scale of ERAS. There has been loss of practitioner engagement, with teams meeting less frequently and not reviewing data or taking action on areas for improvement, especially postoperative complications and postoperative adherence elements, many of which could be considered standard of care. Consequently, there has been no appreciable increase in ERAS adherence, indicating that ERAS is possibly in a sustainment phase. However, sustainment should not be entertained within AHS until at least an overall adherence of 75% to 80% is achieved; therefore, more frequent ERAS team meetings are required to review ERAS adherence and outcomes combined with PDSA cycles targeted at problem areas of low adherence.^28^

The overall adherence in the ERAS gynecologic oncology cohort was 75%. This was associated with an LOS reduction of 0.6 days overall, with the findings most pronounced in the high-complexity (debulking) group in which a 1.5-day reduction in LOS was observed. These findings are in keeping with level I studies^29,30^ that supported the role of ERAS in ovarian cancer debulking, despite the fact that a recent survey^31^ of gynecologic oncologists found low ERAS uptake in cytoreductive surgery. Like colorectal surgery, a dose-response association has been identified between ERAS adherence and outcomes within gynecologic oncology surgery. Wijk et al^32^ found that every unit increase in ERAS guideline score was associated with a 12% decrease in LOS among high-complexity gynecologic oncology surgery. Similar to colorectal surgery, Pache et al^33^ found that an overall ERAS adherence greater than 70% was associated with a shorter LOS in gynecologic oncology.

Although an LOS reduction was not observed within the ERAS liver cohort, a 73% reduction in serious complications was found. A recent meta-analysis^34^ of ERAS programs in liver surgery found that ERAS was associated with a 2-day reduction in LOS and 29% reduction in complications; however, the included studies were noted to be heterogeneous. Not all studies have found benefit with ERAS in liver surgery, including the study by Pickens et al,^24^ which found no improvement in LOS or complications. This finding may be attributed to the fact that no clinically significant increase in ERAS liver protocol adherence was reported in their study. The lack of improvement in LOS in the current study may be associated with the fact that only a modest increase in ERAS liver protocol adherence of 8% was observed.

Despite a greater than 20% increase in adherence, this study found that the ERAS pancreas cohort had a mean adjusted LOS increase of 2.8 days. It is difficult to know why this occurred other than to speculate that pancreatic surgery is especially complex and to date several studies have failed to find benefit with ERAS implementation. Pickens et al^24^ found no change in LOS in their ERAS pancreatic surgery cohort but in fact found a 12% increase in 30-day complications. Roulin et al^35^ concluded that implementation of ERAS in pancreatoduodenectomy is challenging, particularly in the postoperative period. They found that an overall pancreatic protocol adherence of 70% or higher was associated with decreased complications and LOS. Of note, in the current study, overall adherence in the ERAS pancreas cohort was 66.0%, and the postoperative adherence rate was only 41.0%. It is likely that further improvements are necessary to see success with ERAS in this challenging patient population.

Although the current study found a reduction in median LOS of 3 days for radical cystectomy on unadjusted analysis, this finding did not remain significant after adjustment. However, a significant finding was found whereby in patients undergoing radical cystectomy who required readmission, the ERAS patients were admitted for 3.4 fewer days than the non-ERAS patients. This is an important finding given that readmission after radical cystectomy may be as high as 30% within 90 days after surgery.^36^

Finally, it is important to evaluate surgical quality improvement initiatives from the standpoint of not only clinical outcome improvements but also cost savings. In a recent health economic analysis^7^ of these ERAS pathways (colorectal, gynecologic oncology, liver, pancreas, and radical cystectomy) within AHS, the return on investment ratio was as high as 7.3, meaning that every dollar invested in ERAS brought $7.3 in return, allowing us to conclude that the ERAS multiguideline implementation was cost-saving in Alberta.

### Strengths and Limitations

This study has strengths and limitations. One strength is the substantial size of the cohort, collected across multiple hospital sites and surgical areas during 5 years of implementation within 1 health care system. Data collected within a consistent context illustrating progressive expansion of ERAS guidelines across surgical areas provide a real-life scenario to examine the potential for ERAS pathways to improve patient and health care system outcomes. This approach may benefit organizations that are considering similar ERAS implementation processes.

The study also has limitations, including its retrospective design and inherent biases. Furthermore, low frequency of results within specific surgical areas may have limited the ability to sufficiently examine significance. This study may have had false-positive findings because of multiple comparisons of data. In addition, although we used all available data to account for confounding by patient characteristics, the pre-post design and the lack of randomized assignment mean that unmeasured variables could explain the association between ERAS and improved outcomes.

## Conclusions

As with most quality improvement initiatives, opportunities for continued improvement remain. Although this study found system-level improvement in ERAS guideline adherence, LOS, and readmissions, some surgical areas improved substantially more than others. These areas will be targeted as part of our ongoing provincial surgical quality improvement mandate, with specific goals of better understanding of practitioner reengagement, team process for advancing improvements, and their association with increasing ERAS adherence above minimum thresholds. In doing so, these efforts can then be translated into benefits for patients, practitioners, and the health care system across multiple surgical disciplines.

## Notes

Supplement. **eAppendix 1.** ERAS Standards **eAppendix 2.** List of Common Compliance Elements Used to Calculate Compliance for Multiple Pathways **eAppendix 3.** Surgery Parameters **eTable.** Variables Selected for Regression Models for Length of Stay
