# Supplementary material for: Evaluation of the Implementation of Multiple Enhanced Recovery After Surgery Pathways Across a Provincial Health Care System in Alberta, Canada
Source: JAMA Netw Open. 2021 Aug 6;4(8):e2119769. doi: 10.1001/jamanetworkopen.2021.19769 (PMC8346943; doi:10.1001/jamanetworkopen.2021.19769)
Supplement: Supplement. — eAppendix 1. ERAS Standards eAppendix 2. List of Common Compliance Elements Used to Calculate Compliance for Multiple Pathways eAppendix 3. Surgery Parameters eTable. Variables Selected for Regression Models for Length of Stay [file jamanetwopen-e2119769-s001.pdf]

Supplementary Online Content

Nelson G, Wang X, Nelson A, et al. Evaluation of the implementation of multiple Enhanced Recovery After Surgery pathways across a provincial health care system in Alberta, Canada. *JAMA Netw Open*. 2021;4(8):e2119769. doi:10.1001/jamanetworkopen.2021.19769

eAppendix 1. ERAS Standards

eAppendix 2. List of Common Compliance Elements Used to Calculate Compliance for Multiple Pathways

eAppendix 3. Surgery Parameters

eTable. Variables Selected for Regression Models for Length of Stay

This supplementary material has been provided by the authors to give readers additional information about their work.

© 2021 Nelson G et al. *JAMA Network Open.*

Appendix 1a

eAppendix. ERAS Standards

1a. ERAS Colorectal Care Compliance Standards, Alberta Health Services, 2013-2018

**Compliance element** based on ERAS Interactive Audit System	**Applicability to Surgery types** based on ERAS Interactive Audit System	**Compliance criteria** based on ERAS Interactive Audit System	**ERASAlberta Recommendation** see AHS *Provincial Clinical Knowledge Topic: ERAS Colorectal Surgery, Adult – Inpatient* https://www.albertahealthservices.ca/cgv/Page15919.aspx (select E)
**Pre-admission phase**			
**Pre-operative nutritional status assessment**	All	Yes, compliant if assessed with Canadian Nutrition Screening Tool (CNST) or Malnutrition Screening Tool (MST)	**Pre-operative optimization: nutritional status** Patients should be screened for nutritional status including weight loss within the previous 6 months. All patients at nutrition risk need an assessment to confirm malnutrition. If a patient is malnourished, an in-depth nutrition assessment, along with treatment, is required by a registered dietitian.
**Pre-operative nutritional treatment**	Only if assessed as at risk or malnourished	Yes, compliant if treated appropriately with either oral supplements, tube feeding, or parenteral nutrition	
**Pre-admission patient education**	All	Yes, compliant if provided	**Pre-operative information, education and counselling** Patients should routinely receive dedicated pre-operative counselling (e.g., regarding activity, drains/ tubes/ lines, and expectations regarding hospital discharge). Additional education for the marking and management of stomas for rectal surgery only.
**Alcohol usage**	Only if drink 3 of more standard alcohol drinks per day	Yes, compliant if stopped alcohol >4 weeks before surgery	**Pre-operative optimization** Pre-operative general medical optimization is necessary before surgery. Smoking should be stopped four weeks before surgery. Alcohol consumption, especially for patients with alcohol dependency, should be stopped four weeks before surgery. Patients with alcohol dependency should wean consumption under the care of a qualified healthcare professional. Increasing exercise pre-operatively may be of benefit.
**Smoker**	Only if smoke >2 cigarettes per day	Yes, compliant if stopped smoking >4 weeks before surgery	
**Pre-operative phase**			
**No oral bowel preparation done, unless applicable**	All (except anterior resection of rectum)	Yes, compliant if no oral bowel preparation	**Pre-operative bowel preparation** Current evidence supports that if mechanical bowel preparation is to be used, it should be in combination with pre-operative oral antibiotics to minimize perioperative infectious complications.

© 2021 Nelson G et al. *JAMA Network Open.*

**Compliance element** based on ERAS Interactive Audit System	**Applicability to Surgery types** based on ERAS Interactive Audit System	**Compliance criteria** based on ERAS Interactive Audit System	**ERASAlberta Recommendation** see AHS *Provincial Clinical Knowledge Topic: ERAS Colorectal Surgery, Adult – Inpatient* https://www.albertahealthservices.ca/cgv/Page15919.aspx (select E)
**Pre-operative oral carbohydrate treatment**	All	Yes, compliant if treatment completed	**Pre-operative fasting and carbohydrate load treatment** Before scheduled procedures, the minimum duration of pre-operative fasting should be 8 hours after a meal that includes meat, fried or fatty foods, 6 hours after a light meal (such as toast and a clear fluid), and 2 hours after clear fluids as per the Canadian Anesthesiologists' Society *Guidelines to the Practice of Anesthesia.* Carbohydrate load treatment should occur between 2 and 3 hours prior to the administration of anesthesia.
**Pre-operative long acting sedative medication**	All	Yes, compliant if not provided	**Pre-anesthetic medication** Patients should not routinely receive long-acting sedative medication before surgery.
**Thrombosis prophylaxis**	All (except patients with high bleeding risk or contraindications to pharmacologic thromboprophylaxis)	Yes, compliant if appropriate medication or appropriate medication, and sequential compression stockings provided, AND first anticoagulant done pre or intraoperatively, AND duration of anticoagulant prophylaxis was the whole length of stay or as an outpatient for 28 days.	**Venous thromboembolism prophylaxis** Patients should have a sequential compression device (SCD) applied, and receive pre-operative or intra-operative pharmacological prophylaxis with heparin. Extended prophylaxis with low molecular weight heparin (LMWH) should be given for an additional 28 days post-discharge to patients with cancer or other patients with increased risk of venous thromboembolism (VTE).
**Antibiotic prophylaxis before incision**	All	Yes, compliant if appropriate medication within specified time provided	**Antimicrobial prophylaxis and skin preparation** Routine prophylaxis using intravenous antibiotics should be given within 60 minutes prior to incision. Additional doses should be given during prolonged operations according to the half-life of the drug used. Surgical site skin preparation with chlorhexidine-alcohol should be used prior to incision.
**PONV prophylaxis administered**	All patients with Apfel score ≥2	Yes, compliant if appropriate medication provided	**Prevention of post-operative nausea and vomiting** All patients need to be pre-operatively assessed for risk and provided with perioperative post-operative nausea and vomiting (PONV) prophylaxis accordingly. A multimodal approach to PONV prophylaxis should be adopted in all high risk patients.
**Intra-operative phase**			
**Upper body forced-air heating cover used**	All	Yes, compliant if used	**Prevention of intra-operative hypothermia** Intra-operative normothermia should be maintained. A suitable warming device and warmed intravenous fluids should be used routinely to keep body temperature >36ºC. Attempts should be made to avoid hypothermia because it increases the risk of perioperative complications.

© 2021 Nelson G et al. *JAMA Network Open.*

**Compliance element** based on ERAS Interactive Audit System	**Applicability to Surgery types** based on ERAS Interactive Audit System	**Compliance criteria** based on ERAS Interactive Audit System	**ERASAlberta Recommendation**<br>see AHS *Provincial Clinical Knowledge Topic: ERAS Colorectal Surgery, Adult – Inpatient*<br>https://www.albertahealthservices.ca/cgv/Page15919.aspx (select E)
**No nasogastric tube used post-operatively**	All	Yes, compliant if not used	**Nasogastric intubation**<br>Post-operative nasogastric tubes should not be used routinely. Nasogastric tubes inserted during surgery should be removed before reversal of anesthesia.
**No epidural or spinal used, unless applicable**	All open surgeries (abdominoperineal resection [APR] contraindicated)<br><br>All minimally invasive surgeries (MIS) or converted (started as MIS)	Open (except APR): Yes, compliant if thoracic epidural analgesia used, no lumbar or caudal epidural analgesia or spinal used, no other reason not used, or if contraindicated<br><br>Open (APR): Yes, compliant if contraindicated<br><br>MIS or converted (except APR): Yes, compliant if epidural analgesia or spinal used, other reason not used, or if contraindicated<br><br>MIS or converted (APR): Yes, compliant if spinal used, other reason not used, or if contraindicated	**Minimally invasive surgery**<br>Minimally invasive surgery (MIS) is recommended for appropriate patients when expertise and resources are available.<br>**Standard anesthetic protocol**<br>Emphasis is placed on using short-acting anesthetic agents with consideration for the use of total intravenous anesthesia (TIVA) instead of inhalation anesthetic. Opioids should be used sparingly and if needed, short-acting opioids are recommended.
**No long-acting systemic opioids given**	All surgeries under general anesthesia	Yes, compliant if long-acting systemic opioids not given or only short acting opioids given (e.g., remifentanil, fentanyl)	
**No resection-site drainage, unless applicable**	All	Yes, compliant if not used	**Surgical site drains**<br>Routine drainage is discouraged because it is an unsupported intervention that is likely to impair mobilization.
**Post-operative phase**			
**Time to termination of urinary drainage (nights)**	All (except rectal procedures; except patients with suprapubic catheter or nephrostomy)	Yes, compliant if removed <48 h (2 nights)	**Urinary drainage**<br>Urinary catheter removal should be considered upon completion of procedure, if appropriate. If required for post-operative bladder drainage, it should be used for a short period only, and removal within 1 to 2 days is recommended. The urinary catheter should be removed regardless of the usage or duration of thoracic epidural analgesia (TEA).
**Duration of IV fluid infusion (nights)**	All	Yes, compliant if discontinued <48 h (2 nights)	**Perioperative fluid management**<br>Very restrictive or liberal fluid regimes should be avoided in favour of euvolemia. The use of advanced hemodynamic monitoring to facilitate individualized fluid therapy during the perioperative period should be considered, especially for high

© 2021 Nelson G et al. *JAMA Network Open.*

**Compliance element** based on ERAS Interactive Audit System	**Applicability to Surgery types** based on ERAS Interactive Audit System	**Compliance criteria** based on ERAS Interactive Audit System	**ERASAlberta Recommendation** see AHS *Provincial Clinical Knowledge Topic: ERAS Colorectal Surgery, Adult – Inpatient* https://www.albertahealthservices.ca/cgv/Page15919.aspx (select E)
			risk patients and patients for which significant intravascular volume loss is anticipated. Balanced crystalloid solutions are preferred to sodium chloride 0.9%. The enteral route for fluid post-operatively should be used as early as possible, and intravenous fluids should be discontinued as soon as clinically appropriate.
**Stimulation of gut motility**	All (except patients with new ileostomy, or ileostomy plus colostomy)	Yes, compliant if stimulant (gum and/ or laxatives) used	**Prevention of post-operative ileus**<br>Thoracic epidural analgesia (TEA) or laparoscopic surgery should be utilized in colonic surgery if possible. Fluid overload and nasogastric decompression should be avoided. A multimodal approach to optimizing gut function should involve chewing gum and oral laxatives.
**Energy intake on day of surgery, post-operatively**	All	Yes, compliant if ≥300 kcal (3 x 90 mL [135 kcal] Ensure Protein Max) consumed POD 0	**Post-operative nutritional care**<br>Patients should be screened for nutritional status and if at risk of under nutrition given active nutritional support. Perioperative fasting should be minimized. Post-operatively patients should be encouraged to take normal food as soon as lucid after surgery. In addition to normal food intake (includes Post-Surgical Transition Diet), patients should be offered oral nutritional supplements (ONS) to maintain adequate intake of protein and energy. An oral ad libitum diet is recommended 4 hours after rectal surgery.
**Energy intake on post-operative day 1**	All	Yes, compliant if ≥600 kcal (5 x 90 mL [135 kcal] Ensure Protein Max) consumed POD 1	
**Balanced fluids day 0**	All	Colonic and small bowel procedures or excision of ileal pouch-anal anastomosis (IPAA) - Yes, compliant if ≤3000 mL total IV volume given<br><br>Rectal procedures except excision of IPAA – Yes, compliant if ≤3500 mL total IV volume given<br><br>Total IV volume includes intra-operative and post-operative IV fluids on day of surgery	**Perioperative fluid management**<br>Very restrictive or liberal fluid regimes should be avoided in favour of euvolemia. The use of advanced hemodynamic monitoring to facilitate individualized fluid therapy during the perioperative period should be considered, especially for high risk patients and patients for which significant intravascular volume loss is anticipated. Balanced crystalloid solutions are preferred to sodium chloride 0.9%. The enteral route for fluid post-operatively should be used as early as possible, and intravenous fluids should be discontinued as soon as clinically appropriate.
**Post-operative epidural used, if applicable**	All open or converted surgeries (ended as open) (APR contraindicated)	Open or converted (except APR): Yes, compliant if thoracic epidural infusion given for >1 night, no lumbar infusion given, no other reason not used, or if contraindicated	**Post-operative analgesia**<br>Perioperative use of multimodal opioid-sparing strategies is recommended. Appropriately placed thoracic epidural analgesia (TEA) using local anesthetics and low-dose opioids should be considered for open surgery.

© 2021 Nelson G et al. *JAMA Network Open.*

**Compliance element** based on ERAS Interactive Audit System	**Applicability to Surgery types** based on ERAS Interactive Audit System	**Compliance criteria** based on ERAS Interactive Audit System	**ERASAlberta Recommendation**<br>see AHS *Provincial Clinical Knowledge Topic: ERAS Colorectal Surgery, Adult – Inpatient* https://www.albertahealthservices.ca/cgv/Page15919.aspx (select E)
	All MIS (APR contraindicated)	Open or converted (APR): Yes, compliant as contraindicated<br>MIS (except APR): Yes, compliant if thoracic epidural infusion given for >1 night, other reason not used, no lumbar infusion given, or if contraindicated<br>MIS (APR): Yes, compliant as contraindicated, or other reason not used	
**Mobilization within post-operative day 1**	All	Yes, compliant if rising from bed to walk or sit in chair POD 0, and/ or walking or sitting in chair ≥4 h POD 1	**Early mobilization**<br>Prolonged immobilization increases the risk of pneumonia, insulin resistance and muscle weakness. Patients should therefore be mobilized. Mobilization to start the evening of post-operative day 0. Patients should be nursed in an environment that encourages independence and mobilization. A care plan that facilitates patients being out of bed for 2 hours on the day of surgery and 6 hours thereafter is recommended.

© 2021 Nelson G et al. *JAMA Network Open.*

Appendix 1b

ERAS Pancreas Care Compliance Standards, Alberta Health Services, 2015-2018

**Compliance element** based on ERAS Interactive Audit System	**Applicability to Surgery types** based on ERAS Interactive Audit System	**Compliance criteria** based on ERAS Interactive Audit System	**ERASAlberta recommendation** see AHS *Provincial Clinical Knowledge Topic: ERAS Pancreas Surgery, Adult – Inpatient* https://www.albertahealthservices.ca/cgv/Page15919.aspx (select E)
**Pre-admission phase**			
**Pre-operative nutritional status assessment**	All	Yes, compliant if assessed with Canadian Nutrition Screening Tool (CNST) or Malnutrition Screening Tool (MST)	**Pre-operative optimization: nutritional status**<br>Patients should be screened for nutritional status including weight loss within the previous 6 months. All patients at nutrition risk need an assessment to confirm malnutrition. If a patient is malnourished, an in-depth nutrition assessment, along with treatment, is required by a registered dietitian. Routine use of pre-operative artificial nutrition is not warranted, but significantly malnourished patients should be optimized with oral supplements or enteral nutrition pre-operatively. All patients undergoing a Whipple or Total Pancreatectomy procedure require registered dietitian consultation.
**Pre-operative nutritional treatment**	Only if assessed as at risk or malnourished	Yes, compliant if treated appropriately with either oral supplements, tube feeding, or parenteral nutrition	
**Pre-admission patient education**	All	Yes, compliant if provided	**Pre-operative information, education and counselling**<br>Patients should receive dedicated pre-operative counselling routinely.<br>**Pre-operative optimization**<br>For patients with alcohol dependency, 1 month of abstinence before surgery is beneficial and should be attempted. Patients with alcohol dependency should wean consumption under the care of a qualified healthcare professional. For daily smokers, 1 month of abstinence before surgery is beneficial. For appropriate groups, both should be attempted. Increasing exercise pre-operatively may be of benefit.
**Alcohol usage**	Only if drink 3 of more standard alcohol drinks per day	Yes, compliant if stopped alcohol >4 weeks before surgery	**Pre-operative optimization**<br>Pre-operative general medical optimization is necessary before surgery. Smoking should be stopped four weeks before surgery. Alcohol consumption, especially for patients with alcohol dependency, should be stopped four weeks before surgery. Patients with alcohol dependency should wean consumption under the care of a qualified healthcare professional. Increasing exercise pre-operatively may be of benefit.
**Smoker**	Only if smoke >2 cigarettes per day	Yes, compliant if stopped smoking >4 weeks before surgery	
**Pre-operative phase**			

© 2021 Nelson G et al. *JAMA Network Open.*

Compliance element based on ERAS Interactive Audit System	Applicability to Surgery types based on ERAS Interactive Audit System	Compliance criteria based on ERAS Interactive Audit System	ERASAlberta recommendation see AHS *Provincial Clinical Knowledge Topic: ERAS Pancreas Surgery, Adult – Inpatient* https://www.albertahealthservices.ca/cgv/Page15919.aspx (select E)
**No oral bowel preparation done, unless applicable**	All	Yes, compliant if no oral bowel preparation	**Pre-operative bowel preparation** Extrapolation of data from studies on colonic surgery and retrospective studies in pancreaticoduodenectomy (PD) show that mechanical bowel preparation (MBP) has no proven benefit. MBP should not be used.
**Pre-operative oral carbohydrate treatment**	All	Yes, compliant if treatment completed	**Pre-operative fasting and carbohydrate load treatment** Before scheduled procedures, the minimum duration of pre-operative fasting should be 8 hours after a meal that includes meat, fried or fatty foods, 6 hours after a light meal (such as toast and a clear fluid), and 2 hours after clear fluids as per the Canadian Anesthesiologists' Society *Guidelines to the Practice of Anesthesia.* Carbohydrate load treatment should occur between 2 and 3 hours prior to the administration of anesthesia.
**Pre-operative long-acting sedative medication**	All	Yes, compliant if not provided	**Pre-anesthetic medication** Data from studies on abdominal surgery show no evidence of clinical benefit from pre-operative use of long-acting sedatives, and they should not be used routinely. Short-acting anxiolytics may be used for procedures such as insertion of epidural catheters.
**Thrombosis prophylaxis**	All (except patients with high bleeding risk or contraindications to pharmacologic thromboprophylaxis)	Yes, compliant if appropriate medication or appropriate medication, and sequential compression stockings provided	**Venous thromboembolism prophylaxis** Patients should have a sequential compression device (SCD) applied, and receive pre-operative or intra-operative pharmacological prophylaxis with heparin. Extended prophylaxis with low molecular weight heparin (LMWH) should be given for an additional 28 days post-discharge to patients with cancer or other patients with increased risk of venous thromboembolism (VTE).
**Antibiotic prophylaxis before incision**	All	Yes, compliant if appropriate medication within specified time provided	**Antimicrobial prophylaxis and skin preparation** Routine prophylaxis using intravenous antibiotics should be given within 60 minutes prior to incision. Additional doses should be given during prolonged operations according to the half-life of the drug used. Surgical site skin preparation with chlorhexidine-alcohol should be used prior to incision.
**PONV prophylaxis administered**	All patients with Apfel score ≥2	Yes, compliant if appropriate medication provided	**Prevention of post-operative nausea and vomiting** All patients need to be pre-operatively assessed for risk and provided with perioperative post-operative nausea and vomiting (PONV) prophylaxis accordingly. A multimodal approach to PONV prophylaxis should be adopted in all high risk patients.
**Intra-operative phase**			

© 2021 Nelson G et al. *JAMA Network Open.*

**Compliance element** based on ERAS Interactive Audit System	**Applicability to Surgery types** based on ERAS Interactive Audit System	**Compliance criteria** based on ERAS Interactive Audit System	**ERASAlberta recommendation** see AHS *Provincial Clinical Knowledge Topic: ERAS Pancreas Surgery, Adult – Inpatient* https://www.albertahealthservices.ca/cgv/Page15919.aspx (select E)
**No epidural or spinal used, unless applicable**	All open surgeries	Open – Yes, compliant if thoracic epidural analgesia used, or if contraindicated	**Surgical approach and incision type**<br>The choice of incision is at the surgeon's discretion, and should be of a length sufficient to ensure good exposure.<br>**Standard anesthetic protocol**<br>Emphasis is placed on using short-acting anesthetic agents with consideration for the use of total intravenous anesthesia (TIVA) instead of inhalation anesthetic. Opioids should be used sparingly and if needed, short-acting opioids are recommended.<br>**Wound catheters and transversus abdominis plane block**<br>Some evidence supports the use of wound catheters or transversus abdominis plane (TAP) blocks in abdominal surgery. Results are conflicting and variable, and mostly from studies on lower gastrointestinal surgery.
**Upper-body forced-air heating cover used**	All	Yes, compliant if used	**Prevention of intra-operative hypothermia**<br>Intra-operative hypothermia should be avoided by using cutaneous warming (i.e., forced-air or circulating-water garment systems).
**No nasogastric tube used post-operatively**	All	Yes, compliant if not used	**Nasogastric intubation**<br>Pre-emptive use of nasogastric tubes post-operatively does not improve outcomes, and their use is not warranted routinely.
**No resection-site drainage, unless applicable**	All	Low risk patient – Yes, compliant if removed early<br><br>High risk patient – Yes, compliant if used for prolonged period	**Surgical site drains**<br>Early removal of drains after 72 hours may be advisable in patients at low risk (i.e., amylase content in drain <5000 U/L) for developing a pancreatic fistula. There is insufficient evidence to recommend routine use of drains, but their use is based only on low-level evidence.
**Post-operative phase**			
**Termination of urinary drainage**	All (except patients with suprapubic catheter or nephrostomy)	Yes, compliant if removed <48 h (2 nights)	**Urinary drainage**<br>If a urinary catheter is required for post-operative bladder drainage, it should be used for a short period, preferably less than 24 hours post-operatively.
**Stimulation of gut motility**	All	Yes, compliant if stimulant (gum and/or laxatives) used	**Prevention of post-operative ileus**<br>A multimodal approach with thoracic epidural analgesia (TEA) and near-zero fluid balance is recommended. Oral laxatives and chewing gum given post-operatively are safe, and may accelerate gastrointestinal transit.

© 2021 Nelson G et al. *JAMA Network Open.*

**Compliance element** based on ERAS Interactive Audit System	**Applicability to Surgery types** based on ERAS Interactive Audit System	**Compliance criteria** based on ERAS Interactive Audit System	**ERASAlberta recommendation** see AHS *Provincial Clinical Knowledge Topic: ERAS Pancreas Surgery, Adult – Inpatient* https://www.albertahealthservices.ca/cgv/Page15919.aspx (select E)
**Post-operative epidural used, if applicable**	All open surgeries with thoracic epidural anesthesia	Yes, compliant if thoracic epidural infusion for >1 night	**Post-operative analgesia**<br>The use of multimodal opioid-sparing strategies is recommended including thoracic epidural analgesia (TEA). When TEA is contraindicated, transversus abdominis plane (TAP) catheters or rectus sheath blocks are acceptable options for post-operative analgesia.
**Balanced fluids day 0**	All	Yes, compliant if ≤3500 mL total IV volume given<br><br>Total IV volume includes intra-operative and post-operative IV fluids on day of surgery	**Perioperative fluid management**<br>Very restrictive or liberal fluid regimes should be avoided in favour of euvolemia. The use of advanced hemodynamic monitoring to facilitate individualized fluid therapy during the perioperative period should be considered, especially for high risk patients and patients for which significant intravascular volume loss is anticipated. The enteral route for fluid post-operatively should be used as early as possible, and intravenous fluids should be discontinued as soon as clinically appropriate.
**Termination of intravenous fluids infusion (duration of IV fluids)**	All	Yes, compliant if discontinued <48 h (2 nights)	
**Mobilization within post-operative day 1**	All	Yes, compliant if rising from bed to walk or sit in chair POD 0, and/ or walking or sitting in chair ≥4 h POD 1	**Early mobilization**<br>Patients should be encouraged to mobilize, starting the evening of post-operative day 0.

© 2021 Nelson G et al. *JAMA Network Open.*

Appendix 1c

ERAS Liver Care Compliance Standards, Alberta Health Services, 2017-2018

**Compliance element** based on ERAS Interactive Audit System	**Applicability to Surgery types** based on ERAS Interactive Audit System	**Compliance criteria** based on ERAS Interactive Audit System	**ERASAlberta Recommendation** see AHS *Provincial Clinical Knowledge Topic: ERAS Liver Surgery, Adult – Inpatient* https://www.albertahealthservices.ca/cgv/Page15919.aspx (select E)
**Pre-admission phase**			
**Pre-operative nutritional status assessment**	All	Yes, compliant if assessed with CNST or MST	**Pre-operative optimization: nutritional status**<br>Patients should be screened for nutritional status including weight loss within the previous 6 months. All patients at nutrition risk need an assessment to confirm malnutrition. If a patient is malnourished, an in-depth nutrition assessment, along with treatment, is required by a registered dietitian.
**Pre-operative nutritional treatment**	Only if assessed as at risk or malnourished	Yes, compliant if treated appropriately with either oral supplements, tube feeding, or parenteral nutrition	
**Pre-admission patient education**	All	Yes, compliant if provided	**Pre-operative information, education and counselling**<br>Patients should receive routine dedicated pre-operative counselling and education before liver surgery.
**Alcohol usage**	Only if drink 3 of more standard alcohol drinks per day	Yes, compliant if stopped alcohol >4 weeks before surgery	**Pre-operative optimization**<br>Pre-operative general medical optimization is necessary before surgery. Smoking should be stopped four weeks before surgery. Alcohol consumption, especially for patients with alcohol dependency, should be stopped four weeks before surgery. Patients with alcohol dependency should wean consumption under the care of a qualified healthcare professional. Increasing exercise pre-operatively may be of benefit.
**Smoker**	Only if smoke >2 cigarettes per day	Yes, compliant if stopped smoking >4 weeks before surgery	
**Pre-operative phase**			
**No oral bowel preparation done, unless applicable**	All	Yes, compliant if <u>no</u> oral bowel preparation	**Pre-operative bowel preparation**<br>Oral mechanical bowel preparation is not indicated before liver surgery.
**Pre-operative oral carbohydrate treatment**	All	Yes, compliant if treatment completed	**Pre-operative fasting and carbohydrate load treatment**<br>Before scheduled procedures, the minimum duration of pre-operative fasting should be 8 hours after a meal that includes meat, fried or fatty foods, 6 hours after a light meal (such as toast and a clear fluid), and 2 hours after clear fluids as per the Canadian Anesthesiologists' Society *Guidelines to the Practice of Anesthesia*. Carbohydrate load treatment should occur between 2 and 3 hours prior to the administration of anesthesia.

© 2021 Nelson G et al. *JAMA Network Open.*

**Compliance element** based on ERAS Interactive Audit System	**Applicability to Surgery types** based on ERAS Interactive Audit System	**Compliance criteria** based on ERAS Interactive Audit System	**ERASAlberta Recommendation** see AHS *Provincial Clinical Knowledge Topic: ERAS Liver Surgery, Adult – Inpatient* https://www.albertahealthservices.ca/cgv/Page15919.aspx (select E)
**Pre-operative long-acting sedative medication**	All	Yes, compliant if not provided	**Pre-anesthetic medication**<br>Long-acting anxiolytic drugs should be avoided before surgery. Short-acting anxiolytics may be used to perform regional analgesia prior to the induction of anesthesia.
**Thrombosis prophylaxis**	All (except patients with high bleeding risk or contraindications to pharmacologic thromboprophylaxis)	Yes, compliant if appropriate medication or appropriate medication, and sequential compression stockings provided	**Venous thromboembolism prophylaxis**<br>Patients should have a sequential compression device (SCD) applied, and receive pre-operative or intra-operative pharmacological prophylaxis with heparin. Extended prophylaxis with low molecular weight heparin (LMWH) should be given for an additional 28 days post-discharge to patients with cancer or other patients with increased risk of venous thromboembolism (VTE).
**Antibiotic prophylaxis before incision**	All	Yes, compliant if appropriate medication within specified time provided	**Antimicrobial prophylaxis and skin preparation**<br>Single dose intravenous antibiotics should be administered before skin incision and less than 1 hour before hepatectomy. Post-operative prophylactic antibiotics are not recommended. Skin preparation with chlorhexidine 2% is superior to povidone-iodine solution.
**PONV prophylaxis administered**	All patients with Apfel score ≥2	Yes, compliant if appropriate medication provided	**Prevention of post-operative nausea and vomiting**<br>All patients need to be pre-operatively assessed for risk and provided with perioperative post-operative nausea and vomiting (PONV) prophylaxis accordingly. A multimodal approach to PONV prophylaxis should be adopted in all high risk patients.
**Pre-operative steroid administration**	All	Yes, compliant if provided for non-diabetic patients<br><br>Yes, compliant if not provided for diabetic patients	**Perioperative steroid administration**<br>There is insufficient evidence to support the routine use of perioperative steroids in patients undergoing liver resection.
**Intra-operative phase**			
**No epidural or spinal used, unless applicable**	All open surgeries	Yes, compliant if spinal used	**Standard anesthetic protocol**<br>Emphasis is placed on using short-acting anesthetic agents with consideration for the use of total intravenous anesthesia (TIVA) instead of inhalation anesthetic. Opioids should be used sparingly and if needed, short-acting opioids are recommended.
**Upper-body forced-air heating cover used**	All	Yes, compliant if used	**Prevention of intra-operative hypothermia**<br>Perioperative normothermia should be maintained during liver resection.

© 2021 Nelson G et al. *JAMA Network Open.*

**Compliance element** based on ERAS Interactive Audit System	**Applicability to Surgery types** based on ERAS Interactive Audit System	**Compliance criteria** based on ERAS Interactive Audit System	**ERASAlberta Recommendation** see AHS *Provincial Clinical Knowledge Topic: ERAS Liver Surgery, Adult – Inpatient* https://www.albertahealthservices.ca/cgv/Page15919.aspx (select E)
**No nasogastric tube used post-operatively**	All	Yes, compliant if not used	**Nasogastric intubation**<br>Prophylactic nasogastric intubation increases the risk of pulmonary complications after hepatectomy. Its routine use is not indicated.
**No long-acting systemic opioids given**	All surgeries under general anesthesia	Yes, compliant if long-acting systemic opioids not given or only short acting opioids given (e.g., remifentanil, fentanyl)	**Standard anesthetic protocol**<br>Emphasis is placed on using short-acting anesthetic agents with consideration for the use of total intravenous anesthesia (TIVA) instead of inhalation anesthetic. Opioids should be used sparingly and if needed, short-acting opioids are recommended.
**No resection-site drainage, unless applicable**	All	Yes, compliant if not used	**Surgical site drains**<br>The routine use of surgical site drains is not recommended.
**Central venous pressure (CVP)**	All	Yes, compliant if <5 cmH_2O	**Perioperative fluid management**<br>The maintenance of low central venous pressure (CVP) (below 5 cmH_2O) with close monitoring during hepatic surgery is advocated. An alternative to monitoring CVP is to monitor stroke volume variation, which should be maintained at 10 to 12% intra-operatively. Balanced crystalloid should be preferred over sodium chloride 0.9% or colloids to maintain intravascular volume and avoid hyperchloremic acidosis or renal dysfunction, respectively.
**Use of 0.9% NaCl**	All	Yes, compliant if not used	
**Type of incision**	All open surgeries	Yes, compliant if median incision, right transverse incision with vertical extension to the xiphoid (J shaped), or subcostal incision extending to the left	**Surgical approach and incision type**<br>The choice of incision is at the surgeon's discretion. It depends on the patient's abdominal shape and location in the liver of the lesion to be resected. Mercedes-type incision should be avoided due to higher incisional hernia risk. Laparoscopic liver resections can be performed by hepato-biliary surgeons experienced in laparoscopic surgery, in particular left lateral sectionectomy and resections of lesions located in anterior segments. There is currently no proven advantage of robotic liver resection in ERAS. Its use should be reserved for clinical trials.<br>**Prevention of delayed gastric emptying**<br>There is insufficient evidence to support the routine use of an omentum flap after left-sided hepatectomy for prevention of delayed gastric emptying (DGE).
**Post-operative phase**			

© 2021 Nelson G et al. *JAMA Network Open.*

**Compliance element** based on ERAS Interactive Audit System	**Applicability to Surgery types** based on ERAS Interactive Audit System	**Compliance criteria** based on ERAS Interactive Audit System	**ERASAlberta Recommendation** see AHS *Provincial Clinical Knowledge Topic: ERAS Liver Surgery, Adult – Inpatient* https://www.albertahealthservices.ca/cgv/Page15919.aspx (select E)
**Termination of urinary drainage**	All (except patients with suprapubic catheter or nephrostomy)	Yes, compliant if removed <48 h (2 nights)	**Urinary drainage**<br>If a urinary catheter is required for post-operative bladder drainage, it should be used for a short period, preferably less than 24 hours post-operatively.
**Termination of IV fluid infusion (duration of IV fluids)**	All	Yes, compliant if discontinued <48 h (2 nights)	**Perioperative fluid management**<br>The enteral route for fluid post-operatively should be used as early as possible, and intravenous fluids should be discontinued as soon as clinically appropriate.
**Energy intake on day of surgery, post-operatively**	All	Yes, compliant if ≥300 kcal (3 x 90 mL [135 kcal] Ensure Protein Max) consumed POD 0	**Post-operative nutritional care**<br>Patients should be encouraged to take normal food as tolerated, as soon as awake and alert after surgery. Oral nutritional supplements (ONS) should be used to supplement total caloric and protein intake.
**Energy intake on post-operative day 1**	All	Yes, compliant if ≥600 kcal (5 x 90 mL [135 kcal] Ensure Protein Max) consumed POD 1	
**Post-operative glycemic control**	All	Yes, compliant if provided	**Post-operative glucose control**<br>Insulin therapy to maintain normoglycemia is recommended.
**Mobilization within post-operative day 1**	All	Yes, compliant if rising from bed to walk or sit in chair POD 0, and/ or walking or sitting in chair ≥4 h POD 1	**Early mobilization**<br>Patients should be encouraged to mobilize, starting the evening of post-operative day 0.

© 2021 Nelson G et al. *JAMA Network Open.*

Appendix 1d

ERAS Gynecologic Oncology Care Compliance Standards, Alberta Health Services, 2016-2018

**Compliance element** based on ERAS Interactive Audit System	**Applicability to Surgery types** based on ERAS Interactive Audit System	**Compliance criteria** based on ERAS Interactive Audit System	**ERASAlberta recommendation** see AHS *Provincial Clinical Knowledge Topic: ERAS Gynecologic Oncology Surgery, Adult – Inpatient* https://www.albertahealthservices.ca/cgv/Page15919.aspx (*select* E)
**Pre-admission phase**			
**Pre-operative nutritional status assessment**	All	Yes, compliant if assessed with Canadian Nutrition Screening Tool (CNST) or Malnutrition Screening Tool (MST)	**Pre-operative optimization: nutritional status** Patients should be screened for nutritional status including weight loss within the previous 6 months. All patients at nutrition risk need an assessment to confirm malnutrition. If a patient is malnourished, an in-depth nutrition assessment, along with treatment, is required by a registered dietitian.
**Pre-operative nutritional treatment**	Only if assessed as at risk or malnourished	Yes, compliant if treated appropriately with either oral supplements, tube feeding, or parenteral nutrition	
**Pre-admission patient education**	All	Yes, compliant if provided	**Pre-operative information, education and counselling** Patients should routinely receive dedicated pre-operative counselling.
**Alcohol usage**	Only if drink 3 of more standard alcohol drinks per day	Yes, compliant if stopped alcohol >4 weeks before surgery	**Pre-operative optimization** Smoking and alcohol consumption (patients with alcohol dependency) should be stopped four weeks before surgery. Patients with alcohol dependency should wean consumption under the care of a qualified healthcare professional. Increasing exercise pre-operatively may be of benefit.
**Smoker**	Only if smoke >2 cigarettes per day	Yes, compliant if stopped smoking >4 weeks before surgery	
**Pre-operative phase**			
**No oral bowel preparation done, unless applicable**	All	Yes, compliant if no oral bowel preparation	**Pre-operative bowel preparation** Mechanical bowel preparation should not be used routinely even when bowel resection is planned.
**Pre-operative oral carbohydrate treatment**	All	Yes, compliant if treatment completed	**Pre-operative fasting and carbohydrate load treatment** Before scheduled procedures, the minimum duration of pre-operative fasting should be 8 hours after a meal that includes meat, fried or fatty foods, 6 hours after a light meal (such as toast and a clear fluid), and 2 hours after clear fluids as per the Canadian Anesthesiologists' Society *Guidelines to the Practice of Anesthesia*. Carbohydrate load

© 2021 Nelson G et al. *JAMA Network Open.*

**Compliance element** based on ERAS Interactive Audit System	**Applicability to Surgery types** based on ERAS Interactive Audit System	**Compliance criteria** based on ERAS Interactive Audit System	**ERASAlberta recommendation** see AHS *Provincial Clinical Knowledge Topic: ERAS Gynecologic Oncology Surgery, Adult – Inpatient* *https://www.albertahealthservices.ca/cgv/Page15919.aspx* (*select* E)
			treatment should occur between 2 and 3 hours prior to the administration of anesthesia.
**Pre-operative long-acting sedative medication**	All	Yes, compliant if not provided	**Pre-anesthetic medication** Patients should not routinely receive long-acting sedative medication before surgery.
**Thrombosis prophylaxis**	All (except patients with high bleeding risk or contraindications to pharmacologic thromboprophylaxis)	Yes, compliant if appropriate medication or appropriate medication, and sequential compression stockings provided	**Venous thromboembolism prophylaxis** Patients should have a sequential compression device (SCD) applied, and receive pre-operative or intra-operative pharmacological prophylaxis with heparin. Patients at risk for venous thromboembolism (VTE) while using systemic estrogen products should be evaluated with consideration for patient risk factors, dose and indication for therapy. If a significant risk is identified, patients may be advised to consider a progestin-only alternative or to stop current therapy prior to the date of surgery. Cessation timing should consider withdrawal bleeding, ovulation, risk of pregnancy, etc. Extended prophylaxis should be provided on the recommendation of a qualified healthcare professional based on the individual risk of the patient.
**Antibiotic prophylaxis before incision**	All	Yes, compliant if appropriate medication within specified time provided	**Antimicrobial prophylaxis and skin preparation** Routine prophylaxis using intravenous antibiotics should be given within 60 minutes prior to incision. Hair clipping is preferred if hair removal is mandatory. Chlorhexidine–alcohol is preferred to aqueous povidone-iodine solution for skin cleansing.
**PONV prophylaxis administered**	All patients with Apfel score ≥2	Yes, compliant if appropriate medication provided	**Prevention of post-operative nausea and vomiting** All patients need to be pre-operatively assessed for risk and provided with perioperative post-operative nausea and vomiting (PONV) prophylaxis accordingly. A multimodal approach to PONV prophylaxis should be adopted in all high risk patients.
**Intra-operative phase**			
**Upper-body forced-air heating cover used**	All	Yes, compliant if used	**Prevention of intra-operative hypothermia** Perioperative normothermia should be maintained. Suitable active warming devices should be used routinely.
**No nasogastric tube used post-operatively**	All	Yes, compliant if not used	**Nasogastric intubation** Routine nasogastric intubation should be avoided. Nasogastric tubes inserted during surgery should be removed before reversal of anesthesia.

© 2021 Nelson G et al. *JAMA Network Open.*

**Compliance element** based on ERAS Interactive Audit System	**Applicability to Surgery types** based on ERAS Interactive Audit System	**Compliance criteria** based on ERAS Interactive Audit System	**ERASAlberta recommendation** see AHS *Provincial Clinical Knowledge Topic: ERAS Gynecologic Oncology Surgery, Adult – Inpatient* https://www.albertahealthservices.ca/cgv/Page15919.aspx (*select* E)
**No long-acting systemic opioids given**	All surgeries under general anesthesia	Yes, compliant if long-acting systemic opioids not given or only short acting opioids given (e.g., remifentanil, fentanyl)	**Standard anesthetic protocol** Emphasis is placed on using short-acting anesthetic agents with consideration for the use of total intravenous anesthesia (TIVA) instead of inhalation anesthetic. Opioids should be used sparingly and if needed, short-acting opioids are recommended.
**No resection-site drainage, unless applicable**	All	Yes, compliant if not used	**Surgical site drains** Peritoneal drainage is not recommended routinely in gynecologic oncology surgery including for patients undergoing lymphadenectomy or bowel surgery.
**Post-operative phase**			
**Time to termination of urinary drainage (nights)**	All (except patients with suprapubic catheter or nephrostomy)	Yes, compliant if removed <48 h (2 nights)	**Urinary drainage** Urinary catheters should be used for post-operative bladder drainage for a short period, preferably <24 hours post-operatively.
**Stimulation of gut motility**	All (except patients with new ileostomy, or ileostomy plus colostomy)	Yes, compliant if stimulant (gum and/or laxatives) used	**Prevention of post-operative ileus** The use of post-operative laxatives and chewing gum should be considered as a multimodal approach to optimizing gut function.
**Duration of IV fluid infusion (nights)**	All	Yes, compliant if discontinued <48 h (2 nights)	**Perioperative fluid management** Very restrictive or liberal fluid regimes should be avoided in favour of euvolemia. In major open surgery and for high risk patients where there is large blood loss (>7 mL/kg) or systemic inflammatory response syndrome (SIRS), the use of advanced hemodynamic monitoring to facilitate individualized fluid therapy and optimize oxygen delivery during the perioperative period is recommended. Intravenous fluids should be terminated within 24 hours after surgery; balanced crystalloid solutions are preferred to sodium chloride 0.9%. The enteral route for fluid post-operatively should be used as early as possible, and intravenous fluids should be discontinued as soon as clinically appropriate.
**Energy intake on day of surgery, post-operatively**	All	Yes, compliant if ≥300 kcal (3 x 90 mL [135 kcal] Ensure Protein Max) consumed POD 0	**Post-operative nutritional care** Patients should be encouraged to take normal food as tolerated, as soon as awake and alert after surgery. Nutrition intake should be initiated post-operatively as soon as possible. Oral nutritional supplements (ONS) should be used to supplement total caloric and protein intake.
**Energy intake on post-operative day 1**	All	Yes, compliant if ≥600 kcal (5 x 90 mL [135 kcal] Ensure Protein Max) consumed POD 1	

© 2021 Nelson G et al. *JAMA Network Open.*

**Compliance element** based on ERAS Interactive Audit System	**Applicability to Surgery types** based on ERAS Interactive Audit System	**Compliance criteria** based on ERAS Interactive Audit System	**ERASAlberta recommendation** see AHS *Provincial Clinical Knowledge Topic: ERAS Gynecologic Oncology Surgery, Adult – Inpatient* *https://www.albertahealthservices.ca/cgv/Page15919.aspx* *(select* E)
**Mobilization within post-operative day 1**	All	Yes, compliant if rising from bed to walk or sit in chair POD 0, and/ or walking or sitting in chair ≥4 h POD 1	**Early mobilization** Patients should be encouraged to mobilize, starting the evening of post-operative day 0.

© 2021 Nelson G et al. *JAMA Network Open.*

Appendix 1e

ERAS Radical Cystectomy Care Compliance Standards, Alberta Health Services, 2016-2018

**Compliance element** based on ERAS Interactive Audit System	**Applicability to Surgery types** based on ERAS Interactive Audit System	**Compliance criteria** based on ERAS Interactive Audit System	**ERASAlberta recommendation** see AHS *Provincial Clinical Knowledge Topic: ERAS Cystectomy Surgery, Adult – Inpatient* https://www.albertahealthservices.ca/cgv/Page15919.aspx (select E)
**Pre-admission phase**			
**Pre-operative nutritional status assessment**	All	Yes, compliant if assessed with Canadian Nutrition Screening Tool (CNST) or Malnutrition Screening Tool (MST)	**Pre-operative optimization: nutritional status** Patients should be screened for nutritional status including weight loss within the previous 6 months. All patients at nutrition risk need an assessment to confirm malnutrition. If a patient is malnourished, an in-depth nutrition assessment, along with treatment, is required by a registered dietitian.
**Pre-operative nutritional treatment**	Only if assessed as at risk or malnourished	Yes, compliant if treated appropriately with either oral supplements, tube feeding, or parenteral nutrition	
**Pre-admission patient education**	All	Yes, compliant if provided	**Pre-operative information, education and counselling** Patients should receive routine dedicated pre-operative counselling and education (e.g., surgical details, hospital stay and discharge criteria in oral and written form, stoma education, patient's expectations).
**Alcohol usage**	Only if drink 3 of more standard alcohol drinks per day	Yes, compliant if stopped alcohol >4 weeks before surgery	**Pre-operative optimization** Pre-operative general medical optimization is necessary before surgery. Smoking should be stopped four weeks before surgery. Alcohol consumption, especially for patients with alcohol dependency, should be stopped four weeks before surgery. Patients with alcohol dependency should wean consumption under the care of a qualified healthcare professional. Increasing exercise pre-operatively may be of benefit.
**Smoker**	Only if smoke >2 cigarettes per day	Yes, compliant if stopped smoking >4 weeks before surgery	
**Pre-operative phase**			
**No oral bowel preparation done, unless applicable**	All	Yes, compliant if no oral bowel preparation	**Pre-operative bowel preparation** Pre-operative bowel preparation can be safely omitted.
**Pre-operative oral carbohydrate treatment**	All	Yes, compliant if treatment completed	**Pre-operative fasting and carbohydrate load treatment** Before scheduled procedures, the minimum duration of pre-operative fasting should be 8 hours after a meal that includes meat, fried or fatty foods, 6 hours after a light meal (such as toast and a clear fluid), and 2 hours after clear fluids as per the Canadian Anesthesiologists' Society

© 2021 Nelson G et al. *JAMA Network Open.*

**Compliance element** based on ERAS Interactive Audit System	**Applicability to Surgery types** based on ERAS Interactive Audit System	**Compliance criteria** based on ERAS Interactive Audit System	**ERASAlberta recommendation** see AHS *Provincial Clinical Knowledge Topic: ERAS Cystectomy Surgery, Adult – Inpatient* https://www.albertahealthservices.ca/cgv/Page15919.aspx (select E)
			Guidelines to the Practice of Anesthesia. Carbohydrate load treatment should occur between 2 and 3 hours prior to the administration of anesthesia.
**No long-acting pre-operative sedative medication**	All	Yes, compliant if not provided	**Pre-anesthetic medication** Patients should not routinely receive long-acting sedative medication before surgery.
**Thrombosis prophylaxis**	All (except patients with high bleeding risk or contraindications to pharmacologic thromboprophylaxis)	Yes, compliant if appropriate medication or appropriate medication, and sequential compression stockings provided	**Venous thromboembolism prophylaxis** Patients should have a sequential compression device (SCD) applied, and receive pre-operative or intra-operative pharmacological prophylaxis with heparin. Extended prophylaxis with low molecular weight heparin (LMWH) should be given for an additional 28 days post-discharge to patients with cancer or other patients with increased risk of venous thromboembolism (VTE).
**Antibiotic prophylaxis before incision**	All	Yes, compliant if appropriate medication within specified time provided	**Antimicrobial prophylaxis and skin preparation** Patients should receive a single dose antimicrobial prophylaxis 1 hour before skin incision. Skin preparation with chlorhexidine-alcohol prevents/ decreases surgical site infection.
**PONV prophylaxis administered**	All patients with Apfel score ≥2	Yes, compliant if appropriate medication provided	**Prevention of post-operative nausea and vomiting** All patients need to be pre-operatively assessed for risk and provided with perioperative post-operative nausea and vomiting (PONV) prophylaxis accordingly. A multimodal approach to PONV prophylaxis should be adopted in all high risk patients.
**Intra-operative phase**			
**No epidural or spinal used, unless applicable**	All open surgeries	Open – Yes, compliant if thoracic epidural analgesia used	**Surgical approach and incision type** Laparoscopic/ robotic cystectomy is not recommended outside a trial setting until long term results are available. **Standard anesthetic protocol** Emphasis is placed on using short-acting anesthetic agents with consideration for the use of total intravenous anesthesia (TIVA) instead of inhalation anesthetic. Opioids should be used sparingly and if needed, short-acting opioids are recommended.
**No long-acting systemic opioids given**	All surgeries under general anesthesia	Yes, compliant if long-acting systemic opioids not given or only short acting opioids given (e.g., remifentanil, fentanyl)	
**Upper-body forced-air heating cover used**	All	Yes, compliant if used	**Prevention of intra-operative hypothermia** Normal body temperature should be maintained perioperatively. Suitable warming devices should be used.
**No nasogastric tube used post-operatively**	All	Yes, compliant if not used	**Nasogastric intubation** Post-operative nasogastric intubation should not be used routinely (early removal is recommended).
**Post-operative phase**			

© 2021 Nelson G et al. *JAMA Network Open.*

**Compliance element** based on ERAS Interactive Audit System	**Applicability to Surgery types** based on ERAS Interactive Audit System	**Compliance criteria** based on ERAS Interactive Audit System	**ERASAlberta recommendation**<br>see AHS *Provincial Clinical Knowledge Topic: ERAS Cystectomy Surgery, Adult – Inpatient* https://www.albertahealthservices.ca/cgv/Page15919.aspx (select E)
**Stimulation of gut motility**	All	Yes, compliant if stimulant (gum and/ or laxatives) used	**Prevention of post-operative ileus**<br>A multimodal approach to optimize gut function should involve gum chewing and oral magnesium.
**Balanced fluids day 0**	All	Yes, compliant if ≤3500 mL total IV volume given<br><br>Total IV volume includes intra-operative and post-operative IV fluids on day of surgery	**Perioperative fluid management**<br>Very restrictive or liberal fluid regimes should be avoided in favour of euvolemia. The use of advanced hemodynamic monitoring to facilitate individualized fluid therapy during the perioperative period should be considered, especially for high risk patients and patients for which significant intravascular volume loss is anticipated. Balanced crystalloid solutions are preferred to sodium chloride 0.9%. The enteral route for fluid post-operatively should be used as early as possible, and intravenous fluids should be discontinued as soon as clinically appropriate.
**Duration of IV fluid infusion (nights)**	All	Yes, compliant if discontinued ≤5 nights	
**Energy intake on post-operative day 1**	All	Yes, compliant if ≥600 kcal (5 x 90 mL [135 kcal] Ensure Protein Max) consumed POD 1	**Post-operative nutritional care**<br>Patients should be allowed a normal diet as tolerated after surgery. They should be cautioned to begin carefully and increase intake according to tolerance over 3 to 4 days. Oral nutritional supplements (ONS) should be used to supplement total caloric and protein intake.
**Energy intake on post-operative day 2**	All	Yes, compliant if ≥600 kcal (5 x 90 mL [135 kcal] Ensure Protein Max) consumed POD 2	
**Mobilization within post-operative day 1**	All	Yes, compliant if rising from bed to walk or sit in chair POD 0, and/ or walking or sitting in chair ≥4 h POD 1	**Early mobilization**<br>Early mobilization should be encouraged. Mobilization to start the evening of post-operative day 0. A care plan that facilitates patients being out of bed for 2 hours on the day of surgery and 6 hours thereafter is recommended.

© 2021 Nelson G et al. *JAMA Network Open.*

eAppendix 2. List of common compliance elements used to calculate compliance for Multiple Pathways.

<u>ERAS care elements common to Multiple Pathways</u>

Pre-admission

- Preoperative nutritional status assessment
- Preoperative nutritional treatment
- Alcohol usage
- Preadmission patient education
- Smoker

Pre-operative

- No Oral bowel preparation done unless applicable
- Preoperative oral carbohydrate treatment
- Preoperative long-acting sedative medication
- Thrombosis prophylaxis
- Antibiotic prophylaxis before incision
- PONV prophylaxis administered

Intra-operative

- Upper-body forced-air heating cover used
- No NG tube used postoperatively (=Nasogastric tube used postoperatively)

Post-operative

- Duration of IV fluid infusion (nights) OR Termination of intravenous fluid infusion (duration of IV fluids)
- Time to termination of urinary drainage (nights) OR Termination of urinary drainage*
- Mobilization within one day

© 2021 Nelson G et al. *JAMA Network Open.*

eAppendix 3. Surgery Parameters

Define 'Procedure complexity' by grouping procedures into '**1 - surgically more complex**' and '**2 - surgically less complex'**. The following are the categories of the 'Procedure name' in EIAS.

**Main procedure name MORE COMPLEX = 1,** LESS COMPLEX = 2	**Group**
**ERAS Colorectal CONFIRMED**	
**9 - Abdominoperineal resection**	**1**
**8 - Anterior resection of rectum**	**1**
**12 - Excision of IPAA**	**1**
3 - Ileocaecal/right hemicol	2
4 - Left hemicolectomy	2
19 - Other large/small bowel surg	2
13 - Other stoma procedures	2
10 - Proctocolectomy	2
11 - Proctocolectomy with anus	2
**7 - Reversal of Hartmann's proc**	**1**
**6 - Sigmoid resection**	**1**
2 - Small bowel resection	2
**5 - Total/Subtotal colectomy**	**1**
**ERAS Liver CONFIRMED**	
**32 - Extended left hemihep**	**1**
**34 - Extended right hemihep**	**1**
**31 - Left hemihepatectomy**	**1**
**40 - Other biliary surgery**	**1**
**35 - Other segmentectomies**	**1**
**33 - Right hemihepatectomy**	**1**
36 - Wedge or minor resections	**2**
**ERAS Pancreas CONFIRMED**	
53 - Distal resection	**2**

© 2021 Nelson G et al. *JAMA Network Open.*

55 - Other pancreatic surgery	**2**
**51 - Pancreatico-duodenectomy**	**1**
**52 - Pylorus-preserv Whipple**	**1**
**56 - Spleno-pancreatectomy**	**1**
**54 - Total pancreatectomy**	**1**
**ERAS Gyne/Onc CONFIRMED**	
**710 – Debulking gynecology**	**1**
711 – Staging gynecology	**2**
**ERAS Cystectomy CONFIRMED**	
**83 - Cystectomy for functional disease (any urinary diversion)**	**1**
**84 - Radical cystectomy without Bricker**	**1**
**85 - Radical cystectomy with ileal conduit (ex: Bricker)**	**1**
**87 - Radical cystectomy with orthotopic bladder substitution (ex: Studer)**	**1**

© 2021 Nelson G et al. *JAMA Network Open.*

eTable. Variables Selected for Regression Models for Length of stay

	Multiple ERAS Pathways		Colorectal		Liver		Pancreas		Gynecologic Oncology		Radical Cystectomy	
**Measure**	**Pre**	**Post**	**Pre**	**Post**	**Pre**	**Post**	**Pre**	**Post**	**Pre**	**Post**	**Pre**	**Post**
**N=**	984	6773	477	5643	99	131	155	286	152	591	101	122
**Age category**	●		●						●		●	
**Sex**					●							
**BMI category**												
**Area of surgery**												
**Procedure group**	●		●		●				●			
**Procedure complexity**	●						●					
**Alcohol usage**	●		●				●		●		●	
**Tobacco usage**											●	
**ASA class**	●		●				●		●			
**Charlson Index**	●		●									
**Diabetes**	●						●				●	
**Final diagnosis**			●								●	
**Site**	●				●				●			

●= Patient Characteristics used in Regression Model identified as significant in Table 1.

© 2021 Nelson G et al. *JAMA Network Open.*
